# TaCOLD1 defines a new regulator of plant height in bread wheat

**DOI:** 10.1111/pbi.13008

**Published:** 2018-09-24

**Authors:** Huixue Dong, Suli Yan, Jie Liu, Pan Liu, Jiaqiang Sun

**Affiliations:** ^1^ National Key Facility for Crop Gene Resources and Genetic Improvement Institute of Crop Sciences Chinese Academy of Agricultural Sciences Beijing China

**Keywords:** bread wheat, plant height, TaCOLD1, heterotrimeric G protein

## Abstract

Plant height is among the most important agronomic traits that influence crop yield. However, in addition to the *Rht‐1* alleles, the molecular basis of plant height in bread wheat remains largely unclear. Based on wheat gene expression profiling analysis, we identify a light‐regulated gene from bread wheat, designated as *TaCOLD1*, whose encoding protein is homologous to cold sensor COLD1 in rice. We show that TaCOLD1 protein is localized to the endoplasmic reticulum (ER) and plasma membrane. Phenotypic analyses show that overexpression of a mutated form of TaCOLD1 (M187K) in bread wheat cultivar Kenong199 (*Rht‐B1b*) background resulted in an obvious reduction in plant height. Further, we demonstrate that the hydrophilic loop of TaCOLD1 (residues 178–296) can interact with TaGα‐7A (the α subunit of heterotrimeric G protein) protein but not TaGα‐1B, and the mutation (M187K) in TaCOLD1 remarkably enhances its interaction with TaGα‐7A. Physical interaction analyses show that the C‐terminal region of TaGα‐7A, which is lacking in the TaGα‐1B protein, is necessary for its interaction with TaCOLD1. Intriguingly, the C‐terminal region of TaGα‐7A is also physically associated with the TaDEP1 protein (an atypical Gγ subunit). Significantly, we discover that TaCOLD1 and mTaCOLD1 (M187K) can interfere with the physical association between TaGα‐7A and TaDEP1. Together, this study reveals that TaCOLD1 acts as a novel regulator of plant height through interfering with the formation of heterotrimeric G protein complex in bread wheat and is a valuable target for the engineering of wheat plant architecture.

## Introduction

Bread wheat (*Triticum aestivum*) is a major staple crop worldwide. By the year 2050, the world population is expected to reach 9.3 billion and global demand for bread wheat is increasing. To guarantee global food security, people have been seeking elite agronomic traits of bread wheat to improve its yield. Height reduction has been associated with yield increases and yield stability in a number of different crop species (Peng *et al*., 1999). During the Green Revolution, substantial increase in bread wheat (*T. aestivum*) yield was realized, at least in part, through the introduction of the *Reduced height* (*Rht*)‐*B1b* and *Rht‐D1b* semi‐dwarfing alleles encoding mutant gibberellin response modulators (Li *et al*., 2012; Peng *et al*., 1999; Van De Velde *et al*., 2017; Wu *et al*., 2011). However, in addition to the *Rht1* alleles applied in the Green Revolution (Peng *et al*., 1999), new types of genes that determine bread wheat plant height remain to be identified. Therefore, with the continued growth of world population, it is an urgent need to develop better suited plant height based on the understanding of genetic basis of plant height in bread wheat.

Heterotrimeric G proteins, comprising Gα, Gβ and Gγ subunits, are intracellular membrane‐attached signal transducers and involved in regulating shoot, root and epidermis development, as well as sugar sensing, hormone responsiveness and abiotic and biotic stress tolerance (Botella, 2012; Jones and Assmann, 2004; Jones *et al*., 2011; Perfus‐Barbeoch *et al*., 2004; Urano and Jones, 2014; Urano *et al*., 2016). In *Arabidopsis* genome, there is one Gα (GPA1) (Ma *et al*., 1990), one Gβ (AGB1) (Weiss *et al*., 1994), and three Gγ subunits (Chakravorty *et al*., 2011; Mason and Botella, 2000, 2001). Plant heterotrimeric G‐proteins are known to be involved in a myriad of physiological and developmental processes (Assmann, 2005; Chen, 2008; Jones, 2002; Jones and Assmann, 2004; Urano *et al*., 2013). Previous studies have shown that different subunits of heterotrimeric G proteins regulate crop plant height. For instance, rice *G*α mutants displayed a dwarf phenotype (Fujisawa *et al*., 1999) and maize *G*α gene mutant *compact plant 2* (*ct2*) displayed a shorter stature phenotype (Bommert *et al*., 2013). In addition, rice *DEP1* (*DENSE AND ERECT PANICLES 1*) gene encodes an atypical Gγ subunit, which interacts with both the Gα (RGA1) and Gβ (RGB1) subunits (Sun *et al*., 2014). Loss‐of‐function mutations of *DEP1* in rice, or *DEP1* homolog in barley caused dwarf phenotype (Huang *et al*., 2009; Wendt *et al*., 2016). Moreover, in the case of *TaDEP1*, the *tadep1*‐*aabbdd* mutant wheat plants generated by CRISPR/Cas9‐based genome editing exhibited a dwarf phenotype, suggesting that TaDEP1 is an important regulator of wheat plant architecture (Zhang *et al*., 2016).

In this study, we identify a light‐regulated bread wheat gene, here designated as *TaCOLD1*, encoding a trans‐membrane protein with high similarity to rice cold sensor COLD1 (Ma *et al*., 2015). Transgenic analyses indicate that TaCOLD1 plays an important role in regulating bread wheat plant height. Furthermore, we show that the central hydrophilic loop of TaCOLD1 (TaCOLD1^HL^) can interact with the C‐terminal region of TaGα‐7A protein. Remarkably, the mutation (M187K) in TaCOLD1 could enhance its interaction with TaGα‐7A. Intriguingly, the C‐terminal region of TaGα‐7A can also physically associate with TaDEP1. We reveal that both TaCOLD1 and mTaCOLD1 (M187K) can interfere with the physical association between TaGα and TaDEP1. Thus, this study uncovers a molecular mechanism underlying the modulation of heterotrimeric G protein signalling in controlling bread wheat plant height.

## Results

### Identification and molecular characterization of TaCOLD1 in bread wheat

**Figure 1:**
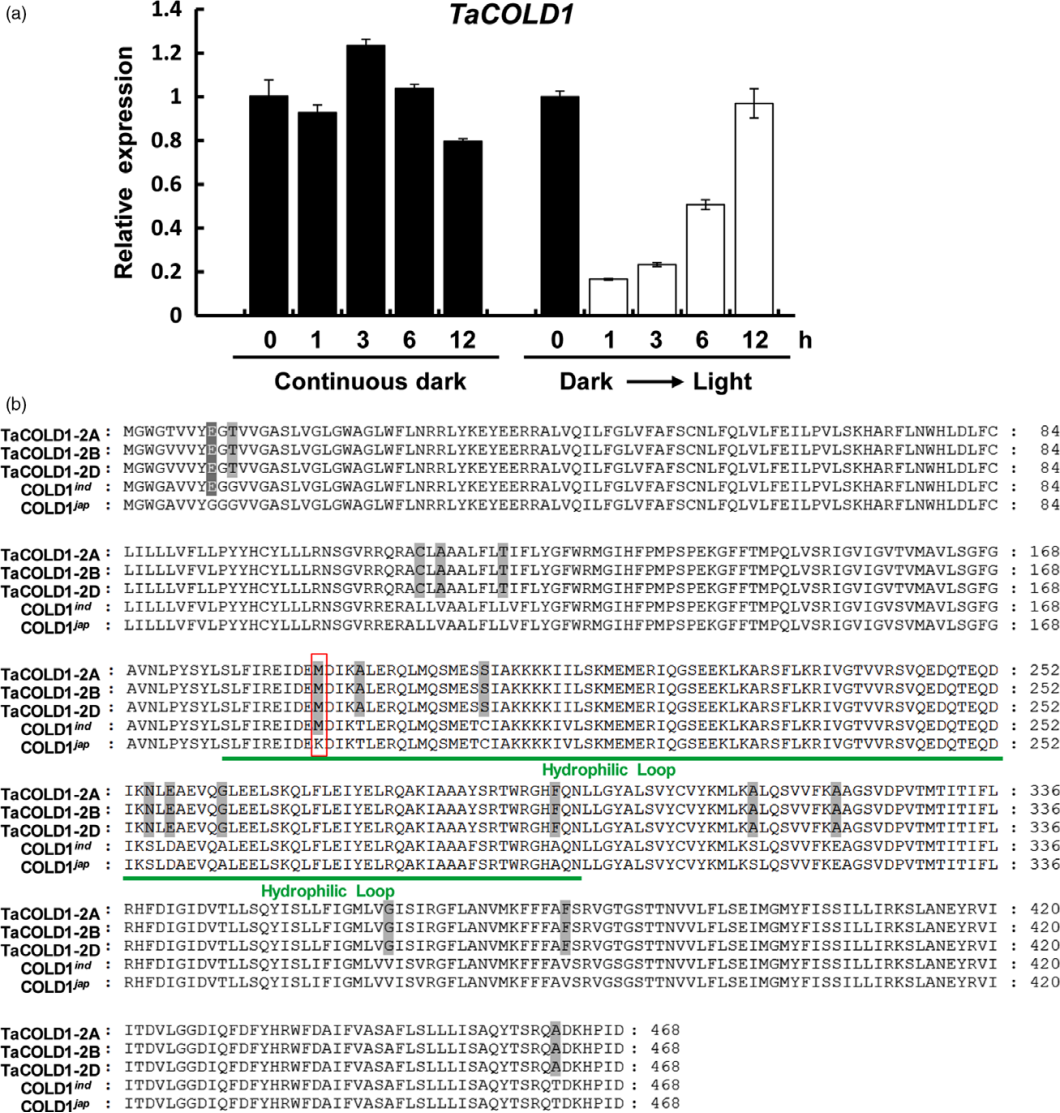
Identification of TaCOLD1 in bread wheat. (a) qRT‐PCR assay showing the expression levels of *TaCOLD1*. The germinated KN199 seedlings were grown in the dark for 5 days (Dark) and then subjected to white‐light irradiation or kept in continuous dark conditions for the indicated time points. The relative expression levels of *TaCOLD1* were normalized to *TaGAPDH*. Error bar represents SD (*n = *3). (b) Alignment of amino‐acid sequences of TaCOLD1‐2A/2B/2D (*Triticum aestivum*), COLD1^*ind*^ (*Oryza. sativa* ssp. *indica*) and COLD1^*jap*^ (*Oryza. sativa* ssp. *japonica*). Green line indicates the hydrophilic loop (residues 178–296). Red box indicates the key SNP (187th) in COLD1. White or grey background represents the identity of amino acid sequences.

**Figure 2:**
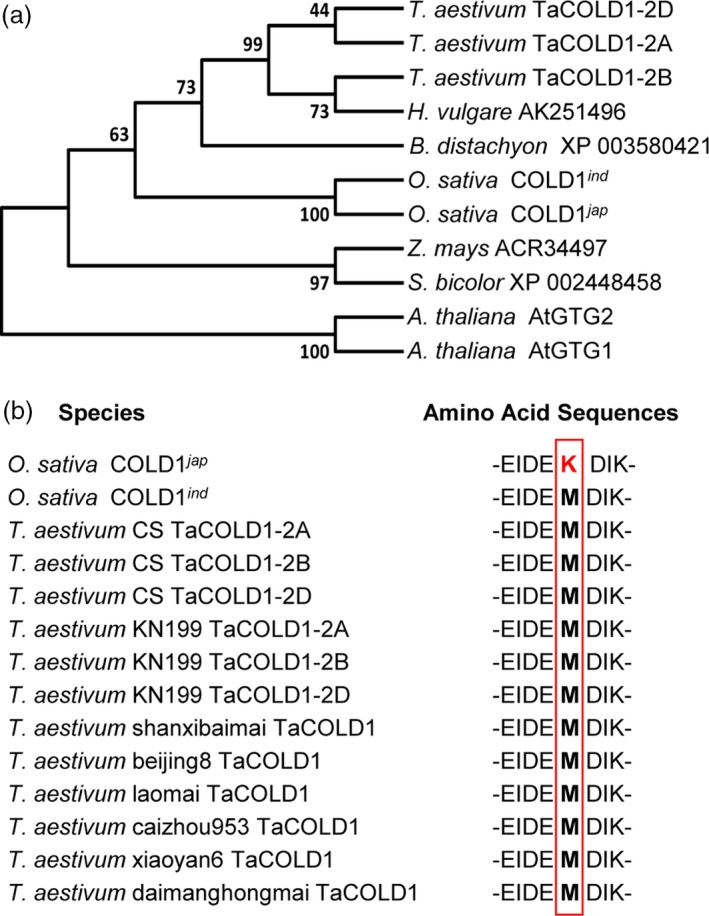
Phylogenetic analysis of TaCOLD1. (a) The phylogenetic tree was constructed based on the neighbour‐joining method by MEGA7 program. The evolutionary distances were computed in units of the number of amino acid substitutions per site. The species and protein names or number are given in the phylogenetic tree. The GenBank accession numbers are MG748865 (TaCOLD1‐2A), MG748866 (TaCOLD1‐2B), MG748867 (TaCOLD1‐2D), XP_015633398 (COLD1^*jap*^), A2XX57 (COLD1^*ind*^), NP_176679 (AtGTG1), NP_194493 (AtGTG2). (b) Amino acid sequence alignment showing the conservation of amino acid Met (marked by red box) in bread wheat varieties.

To identify the regulatory genes for wheat plant architecture, we examined the public wheat expression profiling data (https://wheat.pw.usda.gov/WheatExp/). We noticed that the expression of one wheat gene (*Triticum monococcum* 2AL_7A5AD3700), here designated as *TaCOLD1* (see below for detail information), was significantly repressed by light, indicating that it might play a role in wheat plant photomorphogenesis. To confirm the expression pattern of *TaCOLD1* genes in the hexaploid bread wheat, the 5‐d‐old seedlings of bread wheat cultivar Kenong199 (KN199) grown in the dark were exposed to light or kept in continuous dark conditions for different time points. Consistently, quantitative reverse transcriptase (qRT)‐PCR assay indeed showed that the expression levels of *TaCOLD1* could be reduced in a short time in response to light signal, while no obvious change in continuous dark conditions (Figure 1a). Here, we isolated three highly conserved homologous sequences separately located on chromosomes 2A, 2B and 2D from the hexaploid bread wheat KN199 (Figure S1). Using TaCOLD1 protein sequences as query, we found that the wheat TaCOLD1 proteins are homologs of COLD1 in rice (Ma *et al*., 2015), with 97% identity to the COLD1^*ind*^ (*Oryza sativa* ssp. *indica*) and COLD1^*jap*^ (*Oryza sativa* ssp. *japonica*) proteins (Figure 1b). Then we constructed a neighbour‐joining phylogenetic tree with Clustal W by aligning the protein sequences of TaCOLD1 and their homologs from various plant species. Phylogenetic analysis showed that TaCOLD1 proteins were closely related to their homologs in *Hordeum vulgare* and *Brachypodium distachyon* (Figure 2a).

### TaCOLD1 might be evolutionarily conserved

A recent study identified one SNP in rice *COLD1* gene, which results in Lys^187^ in COLD1^*jap*^ compared to Met^187^ in COLD1^*ind*^, is associated with stronger chilling tolerance of *japonica* cultivars compared to *indica* cultivars (Ma *et al*., 2015), suggesting this amino acid site might be critical for the function of COLD1 homologs. To investigate if there exists a similar SNP in wheat, we performed sequence analyses for the *COLD1* genes from nine accessions of diploid (Qin *et al*., 2017), 22 accessions of tetraploid (Qin *et al*., 2017) and nine accessions of hexaploid wheat. Intriguingly, we found that this locus in all the examined COLD1 proteins is identical to COLD1^*ind*^ but not COLD1^*jap*^ (Figure 2b and Table S1). These results suggest that the wheat COLD1 proteins might be evolutionarily conserved.

### TaCOLD1 is a membrane protein

**Figure 3:**
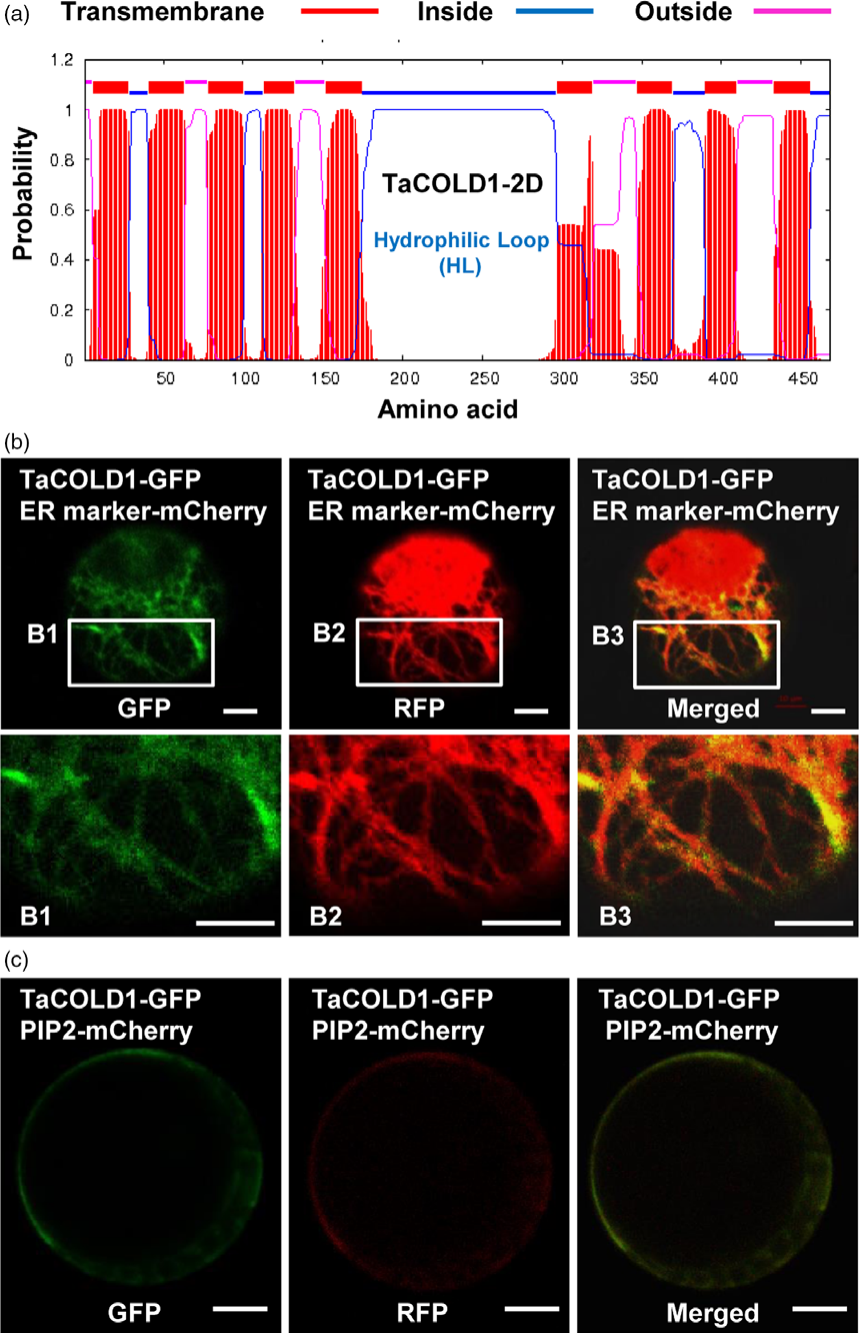
Transmembrane structure and subcellular localization analyses of TaCOLD1. (a) Topology prediction for TaCOLD1‐2D using a transmembrane domain hidden Markov model (TMHMM version 2.0). (b) Subcellular co‐localization of TaCOLD1 with ER marker in bread wheat mesophyll protoplasts. GFP signal of TaCOLD1‐GFP was merged with that of ER marker in the ER. The B1, B2 and B3 images (lower panel) are enlargements of the regions framed in white (upper panel). Scale bars, 10 μm. (c) Plasma membrane localization of TaCOLD1‐GFP. TaCOLD1**‐** GFP signal was merged with that of the PIP2‐mCherry marker at plasma membrane in wheat mesophyll protoplasts.

Prediction of subcellular localization with a transmembrane domain hidden Markov model (Ma *et al*., 2015) suggested that TaCOLD1 proteins were typical transmembrane proteins with nine transmembrane domains (Figures 3a and S2). To experimentally confirm this prediction, the TaCOLD1‐GFP fusion protein and PIP2‐mCherry, a marker for the endoplasmic reticulum (ER) and plasma membrane (Lee *et al*., 2009), were co‐expressed in wheat mesophyll protoplasts. The results showed that the fluorescence signal of TaCOLD1‐GFP could be merged with that of PIP2‐mCherry at ER with a reticular pattern and plasma membrane (Figure 3b,c), suggesting that TaCOLD1 proteins localize to the ER and plasma membrane.

### Overexpression of mTaCOLD1 (M187K) caused a dwarf phenotype in bread wheat

**Figure 4:**
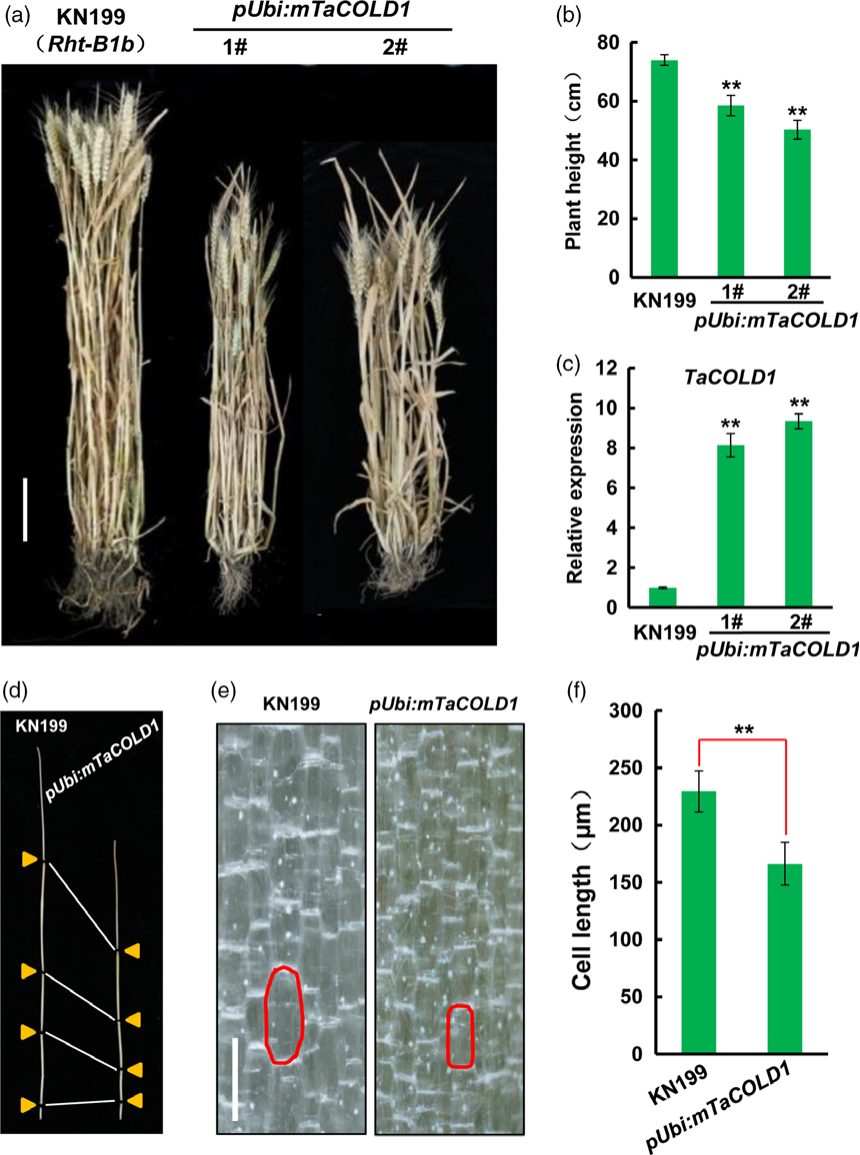
Phenotypic analyses of *pUbi:mTaCOLD1* transgenic bread wheat lines. (a) The whole plant view of WT KN199 (left) and two independent *pUbi:mTaCOLD1* transgenic lines (right) at mature stage. KN199 contains a semi‐dwarf gene *Rht‐B1b*. Scale bar, 10 cm. (b) Statistical analyses of the plant height of WT KN199 and *pUbi:mTaCOLD1* transgenic lines at mature stage. Error bars represent SD (*n *≥* *5). **, *P *<* *0.01 (Student's *t* test). (c) Expression analyses of *TaCOLD1* in WT KN199 and *pUbi:mTaCOLD1* transgenic lines by real‐time RT‐PCR. The transcript levels of *TaCOLD1* were quantified by normalizing against *TaGAPDH*. Mean values of *TaCOLD1* in WT KN199 were defined as “1”. Error bars represent SD (*n *=* *3). **,* P *<* *0.01 (Student's *t* test). (d) Comparison of plant stem length between WT KN199 (left) and *pUbi:mTaCOLD1* transgenic wheat line 1# (right). (e) Longitudinal sections of WT KN199 and *pUbi::mTaCOLD1* transgenic wheat line 1# in the uppermost internode. Characteristic cells are marked in red. Scale bar, 200 μm. (f) Statistical analyses of the cell lengths in (e). Error bars denote SD (*n *≥* *40). **,* P *<* *0.01 (Student's *t* test).

To explore the biological role of TaCOLD1 in bread wheat, we generated the mTaCOLD1 (M187K, identical to COLD1^*jap*^) transgenic lines in bread wheat cultivar KN199 background which already contains a semi‐dwarf gene *Rht‐B1b* (Figure S3). Significantly, the different mTaCOLD1 transgenic wheat lines displayed reduced plant height compared with WT KN199 at both vegetative and mature stages (Figures 4a and S4). Statistical analyses showed that a dramatic decrease of plant weight from ~70 cm in WT KN199 to ~50 cm in transgenic lines (Figure 4b). Consistently, qPCR confirmed that the expression levels of *mTaCOLD1* were obviously increased in the mTaCOLD1 transgenic lines (Figure 4c). Further, each internode of mTaCOLD1 transgenic plants was shorter than that in WT KN199 (Figure 4d). Therefore, we propose that TaCOLD1 may be a critical regulator of plant height in bread wheat.

To investigate the dwarf phenotype of mTaCOLD1 transgenic lines in the cell level, we observed the longitudinal sections of KN199 and mTaCOLD1 transgenic lines using the uppermost internodes at vegetative stage. The cell length in mTaCOLD1 transgenic plants was greatly shorter compared with that in WT KN199 (Figure 4e,f). Taken together, we conclude that overexpression of mTaCOLD1 leads to a dwarf phenotype in bread wheat, at least in part, through reducing cell length.

### Identification of distinct Gα proteins in bread wheat

**Figure 5:**
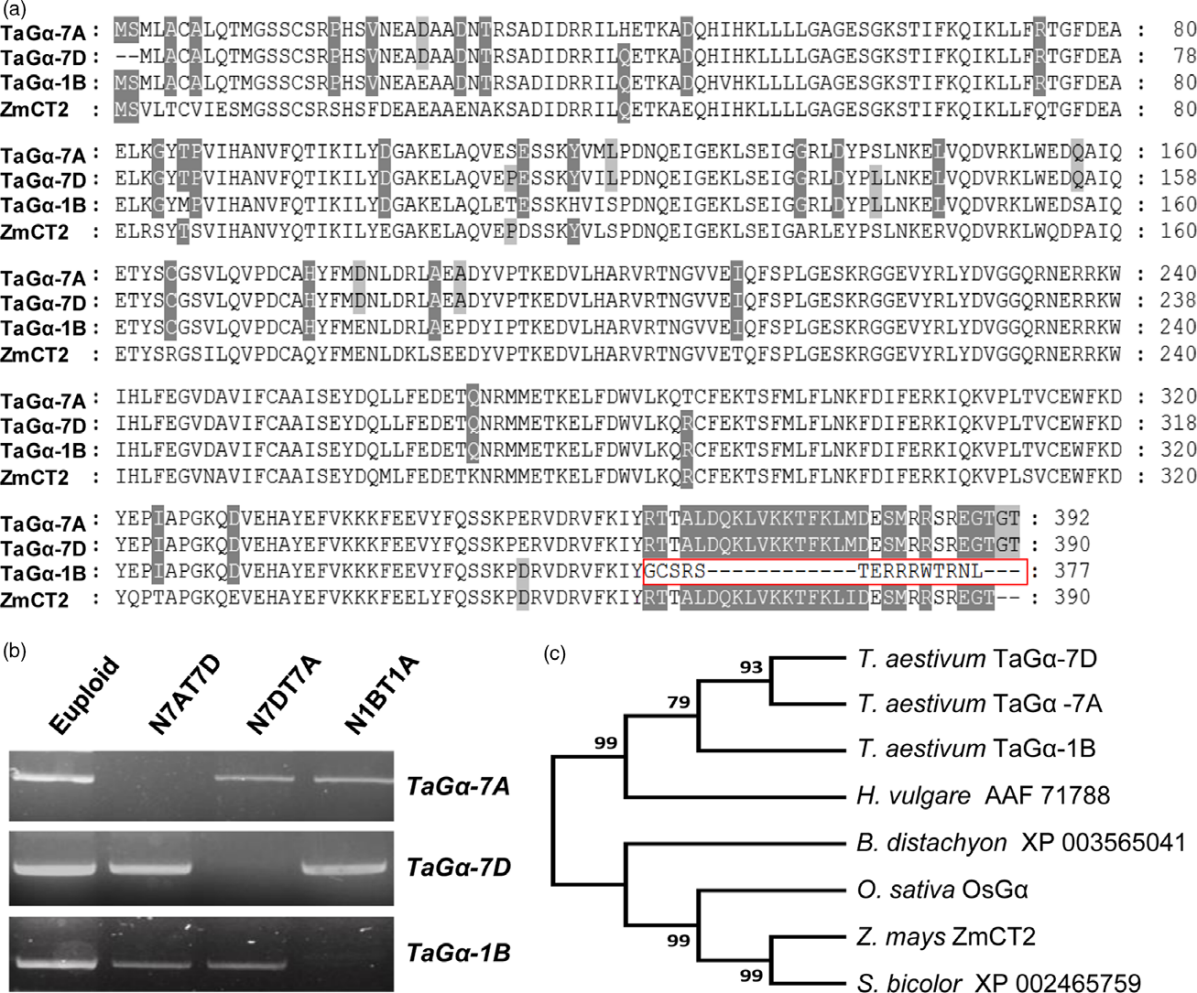
Identification and phylogenetic analysis of distinct TaGα proteins. (a) Alignment of the amino‐acid sequences of TaGα‐7A/7D/1B (*Triticum aestivum*) and ZmCT2 (*Z. mays*). Red rectangle indicates that the C‐terminal region of TaGα‐7A/7D is lacking in the TaGα‐1B protein. White or gray background represents the identity of amino acid sequences. (b) Chromosome location analyses using Chinese Spring nullisomic‐tetrasomic lines. Electrophoretogram of gene *TaG*α from wheat lines lacking individual group 7 and group 1 chromosomes (nullisomic 7A‐tetrasomic 7D, N7AT7D; nullisomic 7D‐tetrasomic 7A, N7DT7A; nullisomic 1B‐tetrasomic 1A, N1BT1A), and euploid control. (c) Phylogenetic analysis of TaGα by MEGA7.0 program. The phylogenetic tree was constructed based on the neighbour‐joining method. The GenBank accession numbers are MG748862 (TaGα‐7A), MG748863 (TaGα‐1B), MG748864 (TaGα‐7D), XP_015639183 (OsGα), NP_001151085 (ZmCT2).

In order to explore the molecular mechanism of TaCOLD1, we planned to identify the partner proteins of TaCOLD1 in bread wheat. A recent study has shown that the homolog of TaCOLD1 in rice, COLD1, is physically associated with Gα protein (Ma *et al*., 2015). Moreover, the maize *COMPACT PLANT2* (*CT2*) gene encodes the α‐subunit (Gα) of heterotrimeric G protein, whose loss‐of‐function mutant displays a shorter stature phenotype (Bommert *et al*., 2013). These findings promoted us to ask whether TaCOLD1 is functionally associated with the heterotrimeric G protein in bread wheat. To test this idea, we first identified the *TaG*α genes based on the coding sequence (CDS) of *ZmCT2* (LOC_Zm00001d027886) in maize and genome sequences of the bread wheat A and D genome donors (Jia *et al*., 2013; Ling *et al*., 2013). Then we isolated three homologous genes from bread wheat cultivar KN199 (Figure S5). Further, chromosomal locations of *TaG*α genes were determined by using the three *TaG*α sequences as query sequences to blast the wheat survey sequences, which include the chromosome‐based draft sequence of the hexaploid wheat (http://wheat-urgi.versailles.inra.fr/blast) (Deng *et al*., 2007; International Wheat Genome Sequencing Consortium, 2014). The results showed that the three *TaG*α sequences were located on chromosomes 7AS, 7DS and 1BL, respectively, and here designated as *TaG*α*‐7A*,* TaG*α*‐7D* and *TaG*α*‐1B*. Protein sequences alignment showed that TaGα‐7A/7D/1B and ZmCT2 protein have ~93% identity (Figure 5a).

We next used the Chinese Spring nullisomic‐tetrasomic lines to experimentally confirm the chromosomal locations of *TaG*α*‐7A*,* TaG*α*‐7D* and *TaG*α*‐1B*. The results showed that target fragments produced by the *TaG*α*‐7A*‐specific primers could not be detected in the absent of chromosome 7A (N7AT7D) (Figure 5b). Similarly, target fragments produced by the *TaG*α*‐7D*‐specific primers could not be detected in the absent of chromosome 7D (N7DT7A); target fragments produced by the *TaG*α*‐1B*‐specific primers could not be detected in the absent of chromosome 1B (N1BT1A) (Figure 5b). In addition, phylogenetic analysis of the Gα proteins from various plant species showed that the three TaGα proteins were most closely related to their homolog in *Hordeum vulgare* (Figure 5c).

### The central hydrophilic loop of TaCOLD1 interacts with TaGα‐7A but not TaGα‐1B

**Figure 6:**
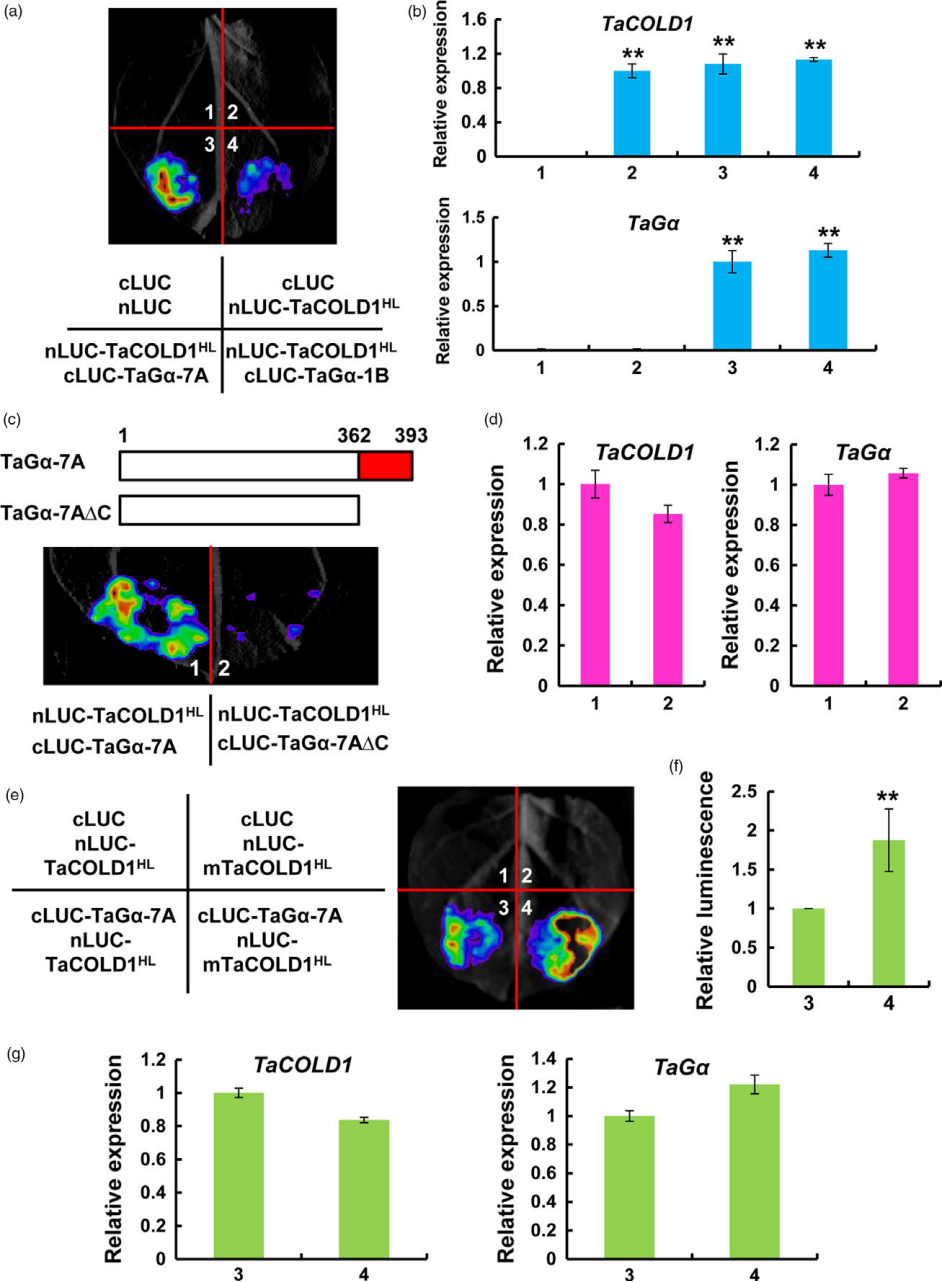
TaCOLD1 physically interacts with TaGα‐7A but not TaGα‐1B. (a) LCI assay demonstrating that TaCOLD1 could directly interact with TaGα‐7A but not TaGα‐1B in *N. benthamiana*. (b) The expression levels of *TaCOLD1* and *TaG*α in the infiltrated *N. benthamiana* leaf areas were determined by qRT‐PCR (mean ± SD,* n* = 5). Results were normalized to *NbACTIN1* (*NbACT1*). **, *P *<* *0.01 (Student's *t* test). (c) LCI assay showing that the C terminus of TaGα‐7A was essentially required for the interaction with TaCOLD1. Upper panel, schematic representation of the truncated TaGα‐7A proteins used for the LCI assays. The full coding sequence of TaGα‐7A protein contains 1‐393 amino acids, while the truncated TaGα‐7A∆C protein contains 1‐362 amino acids. Lower panel, a representative image of LCI assay showing that TaCOLD1^HL^ interacts with TaGα‐7A but not TaGα‐7A∆C in *N. benthamiana*. (d) qRT‐PCR determination of the expression levels of *TaCOLD1* and *TaG*α in the infiltrated *N. benthamiana* leaf areas shown in (c). The data were normalized to *NbACT1*. (Mean± SD,* n* = 4). (e) LCI assay indicating that mTaCOLD1 enhances the interaction with TaGα‐7A. (f) Quantification of the relative luminescence intensities shown in (e), (*n *=* *16). The values in combination 3 were defined as “1”. Error bars indicate SD among three independent replicates. **, *P *<* *0.01 (Student's *t* test). (g) qRT‐PCR assay showing the expression levels of *TaCOLD1* and *TaG*α in the infiltrated *N. benthamiana* leaves shown in (e). The data were normalized to *NbACT1*. (Mean± SD,* n* = 4). In (a), (c) and (e), four independent tobacco leaves were used for the assays and three independent biological replications were performed with similar results.

To determine whether TaCOLD1 proteins physically interact with distinct TaGα proteins, we performed firefly luciferase complementation imaging (LCI) assays in *Nicotiana benthamiana* leaves. The central hydrophilic loop (HL, residues 178–296) of TaCOLD1 was selected for the physical interaction assay, which was fused with the nLUC to generate nLUC‐TaCOLD1^HL^. Meanwhile, TaGα‐7A and TaGα‐1B were fused with the cLUC to generate cLUC‐TaGα‐7A and cLUC‐TaGα‐1B, respectively. Interestingly, strong luminescence signals were observed in nLUC‐TaCOLD1^HL^/cLUC‐TaGα‐7A co‐expression samples and weak luminescence signals were observed in nLUC‐TaCOLD1^HL^/cLUC‐TaGα‐1B co‐expression samples, whereas no signal was detected in the negative controls (Figure 6a). Meanwhile, our qRT‐PCR assays revealed that *TaCOLD1* and *TaG*α were similarly expressed in different infiltrated samples (Figure 6b). Notably, we found that the C‐terminal region of TaGα‐7A is lacking in TaGα‐1B (Figure 5a). To assess whether the C‐terminal region of TaGα‐7A is necessary for its physical interaction with TaCOLD1^HL^, the truncated TaGα‐7A protein without C‐terminal region (TaGα‐7A∆C) was fused with the cLUC to generate cLUC‐TaGα‐7A∆C for LCI assays (Figure 6c). The results showed that obvious LUC activities were detected in nLUC‐TaCOLD1^HL^/cLUC‐TaGα‐7A co‐expression samples, but no LUC activity was detected in nLUC‐TaCOLD1^HL^/cLUC‐TaGα‐7A∆C co‐expression samples (Figure 6c), which expressed similar transcript levels of *TaCOLD1* and *TaG*α (Figure 6d), confirming that the C‐terminal region of TaGα‐7A is indeed required for the physical interaction between TaCOLD1^HL^ and TaGα. Further, to investigate whether the mutation (M187K) of TaCOLD1 affects its interaction with TaGα‐7A, we performed LCI assay in *N. benthamiana* leaves. The results indicate that the mutation (M187K) of TaCOLD1 enhances its interaction with TaGα‐7A (Figure 6e–g).

**Figure 7:**
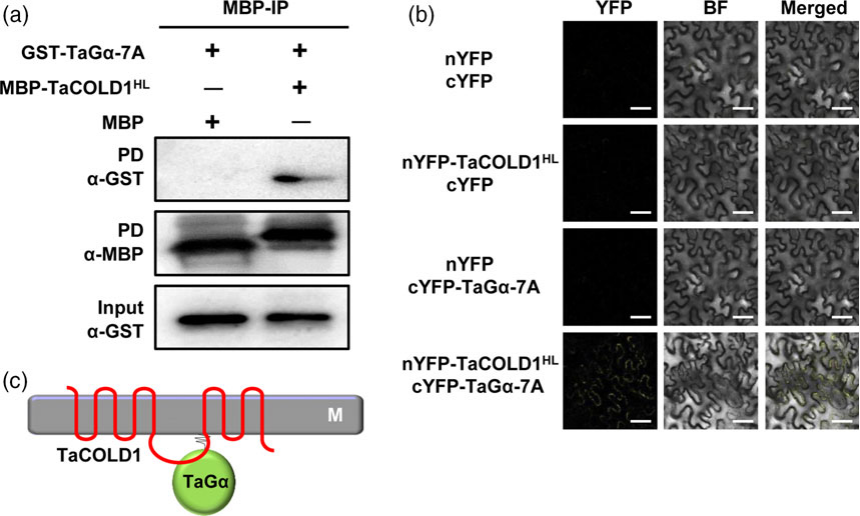
TaCOLD1 interacts with TaGα‐7A *in vitro* and *in vivo*. (a) Pull‐down assay showing the interaction between TaCOLD1 and TaGα. Anti‐MBP and anti‐GST antibodies were used for the immunoblotting. (b) BiFC assay detecting the physical interaction between TaCOLD1 and TaGα‐7A in *N. benthamiana*. The YFP fluorescence signals were detected 48 h post infiltration (hpi). BF, bright field. Scale bars, 10 μm. (c) Schematic showing the interaction between TaCOLD1 and TaGα.

To further evaluate the physical interaction between TaCOLD1 and TaGα‐7A, we performed pull‐down assays *in vitro*. As shown in Figure 7a, GST‐TaGα‐7A was pulled down by MBP‐TaCOLD1^HL^ but not MBP alone. Next, we performed the bimolecular fluorescence complementation (BiFC) assays in *N. benthamiana* to validate the interaction of TaCOLD1^HL^ and TaGα‐7A. The results showed that a strong YFP fluorescence signal was detected on the plasma membrane when nYFP‐TaCOLD1^HL^ was co‐transformed with cYFP‐TaGα‐7A, and no fluorescence signal was detected in the negative controls (Figure 7b). Taken together, these results demonstrate that TaCOLD1 physically interacts with TaGα‐7A but not TaGα‐1B, through the central hydrophilic loop of TaCOLD1 and the C‐terminal region of TaGα‐7A, respectively (Figure 7c).

### TaGα‐7A but not TaGα‐1B physically associates with TaDEP1

A previous study has shown that OsDEP1 in rice functions as an atypical G protein γ subunit (Sun *et al*., 2014). Moreover, a recent study showed that the *tadep1‐aabbdd* wheat mutant, generated by CRISPR/Cas9‐based genome editing, displayed a dwarf phenotype during the vegetative and reproductive growth stages (Zhang *et al*., 2016). These findings promoted us to ask whether TaCOLD1 is functionally associated with TaDEP1. To this end, we first cloned a *TaDEP1* gene from bread wheat cultivar KN199 (Figures S6 and S7). A neighbour‐joining phylogenetic tree showed that TaDEP1 is closely related to HvDEP1 in *Hordeum vulgare* (Figure S8).

**Figure 8:**
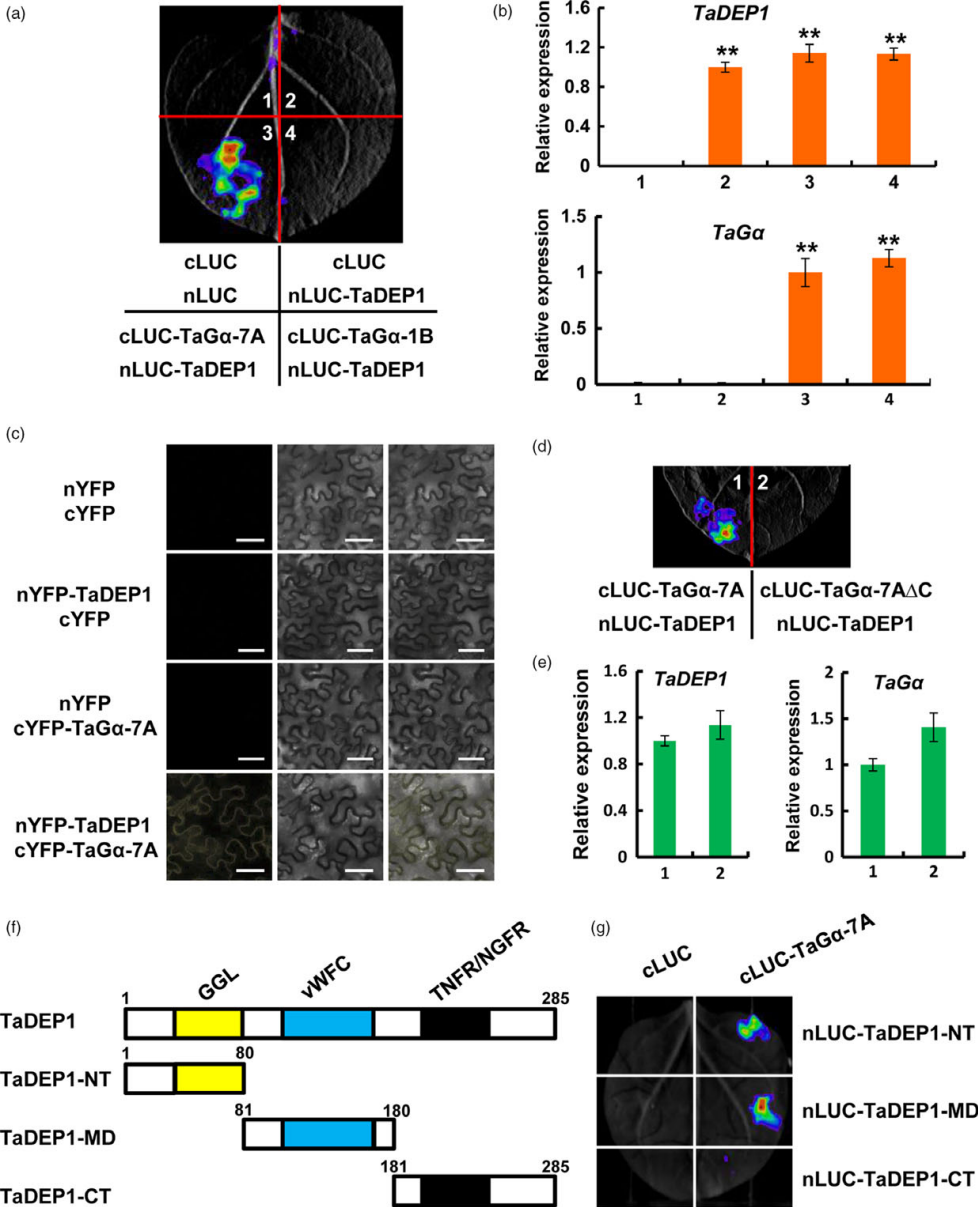
The C‐terminal region of TaGα‐7A physically associates with TaDEP1. (a) LCI assays showing that TaGα‐7A but not TaGα‐1B associates with TaDEP1 in *N. benthamiana* leaves. Empty vectors were used as negative controls. (b) qRT‐PCR assay showing the expression levels of *TaDEP1* and *TaG*α in the infiltrated *N. benthamiana* leaves (mean± SD,* n* = 5). Results were normalized to *NbACT1*. **, *P* < 0.01 (Student's *t* test). (c) BiFC assay demonstrating the physical association between TaDEP1 and TaGα‐7A in *N. benthamiana*. The YFP fluorescence signals were detected 48 h post infiltration (hpi). BF, bright field. Scale bars, 50 μm. (d) LCI assay showing that the C‐terminal region of TaGα‐7A is required for its association with TaDEP1. (e) qRT‐PCR assay showing the expression levels of *TaDEP1* and *TaG*α in the infiltrated *N. benthamiana* leaf areas shown in (d). The data were normalized to *NbACT1*. (Mean± SD,* n* = 5). (f) Schematic representation of the truncated TaDEP1 proteins used for the LCI assays. The *N* terminus of TaDEP1 (TaDEP1‐NT) contains the 1‐80 amino acids; the middle domain of TaDEP1 (TaDEP1‐MD) contains the 81‐180 amino acids; the C terminus of TaDEP1 (TaDEP1‐CT) contains the 181‐285 amino acids. GGL, G protein γ‐like; vWFC, von Willebrand factor type C; TNFR/NGFR, tumor necrosis factor receptor/nerve growth factor receptor. (g) LCI assay showing that the NT and MD domains of TaDEP1 mediate its association with TaGα. Empty vectors were used as negative controls. Three biological replications were performed with similar results.

We hypothesized that TaDEP1, similar to OsDEP1 in rice, acts as a Gγ subunit and might associate with other subunits of G protein in bread wheat. To test this hypothesis, we evaluated the physical association between TaGα and TaDEP1 by LCI assays in *N. benthamiana*. The results showed that the samples co‐expressing cLUC‐TaGα‐7A and nLUC‐TaDEP1 displayed strong luminescence signals, whereas no signal was detected in the samples co‐expressing cLUC‐TaGα‐1B and nLUC‐TaDEP1 (Figure 8a). Our qRT‐PCR assays revealed that the transcript levels of *TaDEP1* and *TaG*α were similar in different infiltrated samples (Figure 8b). To further investigate the physical association between TaGα‐7A and TaDEP1, we performed BiFC assays in *N. benthamiana*. The results showed that an obvious YFP fluorescence signal was detected on the plasma membrane when nYFP‐TaDEP1 was co‐transformed with cYFP‐TaGα‐7A, whereas no fluorescence signal was detected in the negative controls (Figure 8c). Moreover, the truncated TaGα‐7A protein lacking its C‐terminal region (TaGα‐7A∆C; Figure 6c, upper panel) failed to associate with TaDEP1 in the LCI assays (Figure 8d). The qRT‐PCR assays revealed that *TaDEP1* and *TaG*α were similarly expressed in different infiltrated samples (Figure 8e). Thus, we concluded that the C‐terminal region of TaGα‐7A is responsible for its association with TaDEP1.

To map which domain of TaDEP1 is required for its association with TaGα‐7A, we performed the LCI assays in *N. benthamiana* leaves. As shown in Figure 8f, TaDEP1 was divided into three parts: N terminus (residues 1‐80) contains a GGL domain; middle domain (residues 81‐180) contains a vWFC domain and C terminus (residues 181‐285) contains a TNFR/NGFR domain. The three parts of TaDEP1 were fused with nLUC, respectively, for LCI assays. The results showed that obvious LUC activities were observed in cLUC‐TaGα‐7A/nLUC‐TaDEP1‐NT and cLUC‐TaGα‐7A/nLUC‐TaDEP1‐MD co‐expression samples, but no LUC activity was observed in cLUC‐TaGα‐7A/nLUC‐TaDEP1‐CT co‐expression samples (Figure 8g). Taken together, these results showed that the GGL and vWFC motifs of TaDEP1 mediate its association with TaGα‐7A.

### TaCOLD1 interferes with the physical association between TaGα‐7A and TaDEP1

**Figure 9:**
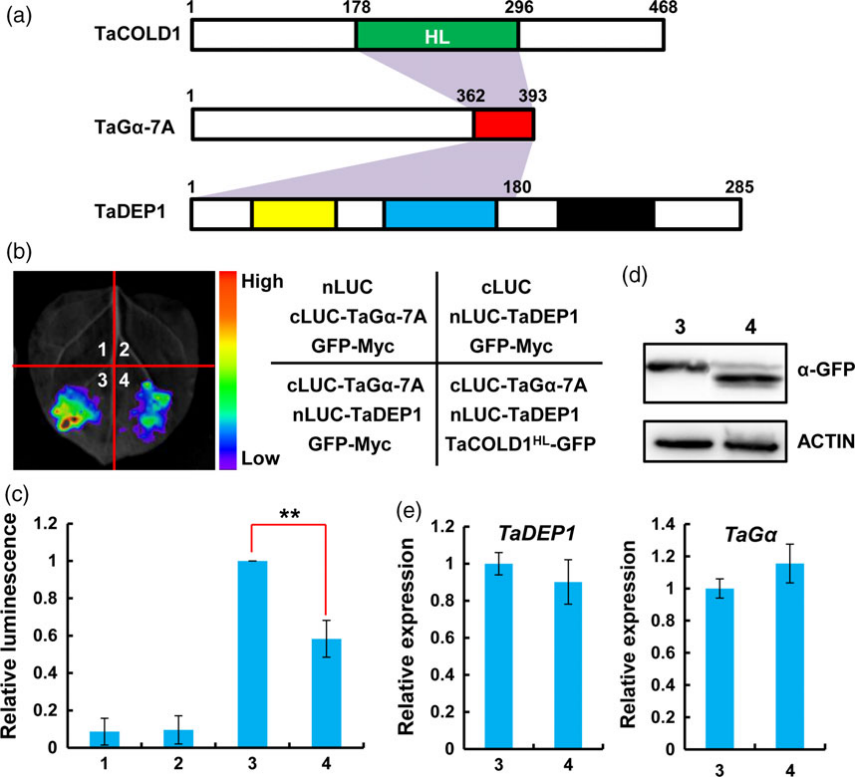
TaCOLD1 interferes with the physical association of TaGα‐7A and TaDEP1. (a) Schematic diagram of the regions required for the associations between TaCOLD1‐TaGα and TaGα‐TaDEP1. (b) LCI assay showing that the association between TaGα‐7A and TaDEP1 was significantly compromised by the co‐expression of TaCOLD1. The representative image was taken at 48 hpi. (c) Quantification of the relative luminescence intensities shown in (b), (*n *=* *18). The values in combination 3 were defined as “1”. Error bars indicate SD among three independent replicates. **, *P *<* *0.01 (Student's *t* test). (d) Immunoblot assay showing the protein levels of TaCOLD1‐GFP and GFP‐MYC in co‐infiltration 3 and 4 in (b). TaCOLD1‐GFP and GFP‐MYC fusion protein levels were determined with anti‐GFP antibody. ACTIN was used as a loading control. (e) qRT‐PCR assay indicating the expression levels of *TaDEP1* and *TaG*α in the infiltrated *N. benthamiana* leaf areas shown in (b). The data were normalized to *NbACT1*. (Mean± SD,* n* = 5).

It has been known that Gα keeps its GDP tightly bound and forms an inactive heterotrimer with the Gβγ‐subunits at steady state (Gilman, 1987). The findings that the C‐terminal region of TaGα‐7A mediates its interaction with both TaCOLD1^HL^ and TaDEP1 (Figures 6c, 8d and 9a), led us to ask whether TaCOLD1 displays a competitive effect on the physical association between TaGα‐7A and TaDEP1. To test this idea, we co‐expressed TaCOLD1^HL^‐GFP with nLUC‐TaDEP1 and cLUC‐TaGα‐7A proteins in *N. benthamiana* leaves. The results showed that the LUC intensities in cLUC‐TaGα‐7A/nLUC‐TaDEP1/TaCOLD1^HL^‐GFP co‐expression samples (Figure 9b,c, co‐infiltration 4) were dramatically decreased by more than 50% compared to those in the cLUC‐TaGα‐7A/nLUC‐TaDEP1/GFP‐MYC co‐expression samples (Figure 9b,c, co‐infiltration 3). Our qRT‐PCR assays showed that *TaDEP1* and *TaG*α were similarly expressed in different infiltrated samples; meanwhile, the immunoblotting assay showed that the protein accumulation levels of TaCOLD1^HL^‐GFP and GFP‐MYC were equal (Figure 9d,e). Similarly, mTaCOLD1 (M187K) could also attenuate the physical association between TaGα‐7A and TaDEP1 (Figure S9). According to these data, we propose that TaCOLD1 might regulate the biological function of heterotrimeric G protein in bread wheat, at least partly, through interfering with the physical association between TaGα‐7A and TaDEP1.

## Discussion

Due to the rapid increasing of world population, food shortage is becoming a serious global problem. Improvement of grain yield has been the key focus of wheat breeding programmes over the past 50 years. Identification of key regulatory genes for important agronomic traits is of enormous significance for wheat molecular assisted breeding. However, map‐based cloning and Genome Wide Association Study (GWAS) in the hexaploid bread wheat with complex genome still remain challenging. In this study, we identify TaCOLD1 as a novel regulator of bread wheat plant height, and uncover a molecular mechanism by which TaCOLD1 might regulate the function of heterotrimeric G protein.

### TaCOLD1 is a novel regulator of plant height in bread wheat

The widely cultivated hexaploid bread wheat (2n = 6x = 42, AABBDD) is derived from the domestication processes, which had been important for the agricultural revolution and the establishment of human civilization. Since the 1970s, green revolution wheat breeding project, characterized by *Rht‐B1b* or *Rht‐D1b* loci, has developed a number of high‐yielding wheat varieties, which are more resistant to damage by wind and rain (Peng *et al*., 1999). Among several pleiotropic functions of *Rht‐B1b*,* Rht‐D1b* and *Rht‐B1c* genes, some are advantageous for plant improvement and some are disadvantageous. For example, *Rht‐B1c* plants can delay heading date and resist to sprouting (Wu *et al*., 2011).

However, other regulators of wheat plant height remain to be identified for ideal wheat plant architecture. In this study, we showed that overexpression of mTaCOLD1 (M187K) can cause an appropriate reduction of plant height in the bread wheat cultivar KN199 containing the *Rht‐B1b* allele (Figures 4a and S4), suggesting that TaCOLD1 acts as a new regulator of bread wheat plant height potentially independent of Rht1.

Therefore, manipulation of *TaCOLD1* orthologues in temperate cereals like bread wheat and barley through genome engineering using the CRISPR/Cas9‐mediated genome editing technology might be applicable to facilitate the breeding of new crop varieties with ideal plant architecture. A haplotype analysis of the critical region of *TaCOLD1* locus (Figure 2b and Table S1) suggested that the *TaCOLD1* gene might have not been used to breed elite wheat varieties.

### A molecular mechanism underlying TaCOLD1 actions in bread wheat

In this study, our results revealed that the central hydrophilic loop of TaCOLD1 could interfere with the formation of heterotrimeric G protein complex in bread wheat. First, we showed that the central hydrophilic loop of TaCOLD1 differentially interacts with distinct TaGα nature variants, such as TaGα‐7A and TaGα‐1B (Figure 6a). Second, domain mapping showed that the C‐terminal region of TaGα‐7A protein is necessary for its association with TaCOLD1 (Figure 6c), whereas this region is lacking in the TaGα‐1B protein. Third, the C‐terminal region of TaGα‐7A protein also mediates its association with TaDEP1 (an atypical Gγ subunit) (Figure 7d). Finally, we uncovered that TaCOLD1 might interfere with the binding of TaGα‐7A and TaDEP1, revealing a molecular mechanism for the TaCOLD1‐mediated regulation of bread wheat plant height. In addition, a recent study reported that the rice COLD1 protein, an orthologue of bread wheat TaCOLD1, could activate Gα GTPase activity (Ma *et al*., 2015). These findings lead us to propose that TaCOLD1 might regulate the function of heterotrimeric G protein through distinct mechanisms in bread wheat.

## Experimental procedures

### Plant materials and growth conditions

Bread wheat (*T. aestivum* L.) cultivar wild‐type (WT) Kenong199 (KN199) was used to amplify gene sequences, generate transgenic wheat plants and analyze gene expression. The mutant *TaCOLD1* gene sequence was ligated to *pUbi:cas* vector and transformed into one‐month‐old embryogenic calli of KN199 by using a PDS1000/He particle bombardment system (Bio‐Rad, Hercules, CA) with a target distance of 6.0 cm from the stopping plate at helium pressure 1100 psi, as described previously (Shan *et al*., 2013).

The WT KN199 and *pUbi:mTaCOLD1* transgenic lines were planted at the experimental station (39°57′N, 116°19′E) of the Institute of Crop Sciences, CAAS, Beijing. The seeds were planted at the beginning of October and harvested in mid‐June next year. Plant heights were measured after harvest.

*Nicotiana benthamiana* were grown in a greenhouse under long‐day conditions (16‐h‐light/8‐h‐dark) at 22 °C.

### Generation of DNA constructs

For LCI assays, the constructs were based on ligation free cloning mastermix (Applied Biological Materials, E011‐5‐A) according to the manufacturer's instruction. In brief, the amplified target genes were separately cloned into the *Kpn*I/*Sal*I digested *p1300‐35S‐nLUC* and *Kpn*I/*Bam*HI digested *p1300‐35S‐cLUC* vectors (Chen *et al*., 2008).

For BiFC assays, gateway cloning technology (Invitrogen, California, CA) was used. All the target genes were ligated into the entry vector *pQBV3* and then introduced into the *pEarleygate201*‐*YN* (nYFP) and *pEarleygate202*‐*YC* (cYFP) destination vectors (Lu *et al*., 2010), respectively.

For pull‐down assays, the constructs were based on the vectors *pMAL‐c2X* and *pGEX4T‐1*. Briefly, the central hydrophilic loop of TaCOLD1 (residues 178–296) was cloned into *Bam*HI/*Sal*I digested *pMAL‐c2X* to generate MBP‐ TaCOLD1^HL^; the coding sequence of TaGα‐7A was cloned into *Eco*RI/*Sal*I digested *pGEX4T‐1* to generate GST‐TaGα‐7A.

The constructs for *35S:TaCOLD1‐GFP* and *35S:TaCOLD1* ^*HL*^ *‐GFP* were based on the gateway cloning technology (Invitrogen). The coding sequence of *TaDEP1* and the central hydrophilic loop of *TaCOLD1* (residues 178–296) were separately ligated into the entry vector *pQBV3* and subsequently introduced into the destination vector *pGWB5* (GFP‐tagged) (Nakagawa *et al*., 2007).

All the primers used for the construction above are summarized in Table S2 and the constructs described above are summarized in Table S4.

### RNA extraction and quantitative RT‐PCR

Trizol reagent (Invitrogen) was used to extract the total RNA of WT KN199 or *pUbi:mTaCOLD1* transgenic lines. About 2 μg of total RNA was applied to synthesize cDNA using the 5× All‐In One RT MasterMix system (Applied Biological Materials) according to the manufacturer's instructions. The cDNA was diluted in 1 : 5 ratios with distilled water, and 2 μL of the diluted cDNA were used as template. SYBR^®^ Premix Ex Taq Kit (TaKaRa, Japan) was used for quantitative RT‐PCR assays in a total volume of 10 μL. Each experiment was repeated with three biological replicates, and each sample was analysed in triplicate. The expression levels of target genes were normalized to *TaGAPDH* or *NbACTIN1* (*NbACT1*). All the primers used for qRT‐PCR assays are listed in Table S3.

### Subcellular localization analysis in bread wheat protoplasts

TaCOLD1‐GFP and a plasma membrane marker, PIP2 (PLASMA MEMBRANE INTRINSIC PROTEIN 2; At3g53420)‐mCherry, were cotransfected into bread wheat mesophyll protoplast cells by the PEG‐mediated method as described previously (Yoo *et al*., 2007). After 20 h‐incubation under dark at 22 °C, protoplasts were examined by a confocal microscopy (Carl Zeiss, LSM880).

### Protein expression and *in vitro* pull‐down assay

The MBP‐TaCOLD1^HL^, GST‐TaGα and MBP proteins were separately expressed in *Escherichia coli* strain BL21 by induction with 0.4 mM isopropyl β‐D‐1‐thiogalactopyranoside (IPTG) at 18 °C overnight, and extracted with the column buffer [20 mM Tris‐HCl, pH 7.4, 200 mM NaCl, 1 mM EDTA, 1 mM PMSF, 1 mM DTT, and 1× protease inhibitor (Roche 4693132001)]. Then, the crude protein extracts of TaGα‐7A were mixed with MBP‐TaCOLD1^HL^ or MBP in equal volume and pulled down using the amylose resin (New England Biolabs) at 4 °C overnight. The amylose resin was washed with column buffer for five times and resuspended in SDS/PAGE loading buffer for immunoblotting assays using the anti‐GST (Cat# CW0144, CWbiotech, Beijing, China) and anti‐MBP (Cat# CW0288, CWbiotech, Beijing, China) antibodies.

### Protein extraction and immunoblotting assays

Total proteins of different infiltrated samples were extracted using the extracted buffer (125 mM Tris‐HCl at pH 6.8, 4% SDS, 0.001% Bromophenol blue, 20% glycerol, 2% β‐Mercaptoethanol). For the immunoblotting detection of TaCOLD1^HL^‐GFP/mTaCOLD1^HL^‐GFP and GFP‐MYC fusion proteins, anti‐GFP (1 : 2000; Roche, 11814460001) and anti‐mouse IgG (1 : 75 000, Sigma, A9044‐2ML) antibodies were used. ACTIN was employed as the loading control with anti‐ACTIN (1 : 5000; CWBIO, CW0264) antibodies.

### Firefly LCI assay

The firefly LCI transient expression assay was performed as previously described (Sun *et al*., 2013). In brief, *Agrobacterium* strain GV3101 carrying the nLUC and cLUC derivative binary plasmids were coinfiltrated in 4‐week‐old *N. benthamiana* leaves. The corresponding empty vectors *p1300‐35S‐nLUC* and *p1300‐35S‐cLUC* were employed as the negative controls. Luciferase activities were measured 48 h after infiltration with the NightSHADE LB 985 (Berthold).

### Bimolecular fluorescence complementation (BiFC) assay

BiFC assay was performed as described previously (Liu *et al*., 2017). Briefly, *Agrobacteria* harbouring the nYFP and cYFP derivative constructs was used together with the *Agrobacterium* p19 strain for infiltration in *N. benthamiana* leaves. The YFP signal was observed by a confocal microscopy (Carl Zeiss, LSM880) after incubation at 22 °C for 48 h.

### Phylogenetic analysis

The homologs of TaCOLD1, TaGα and TaDEP1 were downloaded from NCBI (http://www.ncbi.nlm.nih.gov/). A neighbour‐joining phylogenetic tree was constructed based on 1000 bootstrap replicates by comparing full‐length protein sequences aligned with the Clustal W algorithm within MEGA7.0.

## Accession Numbers

Sequence data from this study can be found in the GenBank database (http://www.ncbi.nlm.nih.gov/) under the following accession numbers: *Rht‐B1b*, MG681100; *TaCOLD1‐2A*, MG748865; *TaCOLD1‐2B*, MG748866; *TaCOLD1‐2D*, MG748867; *TaG*α*‐7A*, MG748862; *TaG*α*‐1B*, MG748863; *TaG*α*‐7D*, MG748864; *TaDEP1*, MG758053.

## Author contributions

J.S. conceived the original screening and research plans; H.D., S.Y., J.L. and P.L. performed the experiments; H.D. and J.S. wrote the article.

## Conflict of interest

The authors have declared that no competing interests exist.

## Supporting information


**Figure S1** The coding sequences of *TaCOLD1‐2A/2B/2D* genes from bread wheat cultivar KN199.
**Figure S2** Topology prediction for TaCOLD1‐2A/2B proteins using a transmembrane domain hidden Markov model (TMHMM version 2.0).
**Figure S3** The coding sequence of *Rht‐B1b* gene from bread wheat cultivar KN199.
**Figure S4** Phenotypes of WT KN199 and *pUbi:mTaCOLD1* transgenic wheat lines grown in the field at vegetative stage.
**Figure S5** The coding sequences of *TaGα‐7A/1B/7D* genes from bread wheat cultivar KN199.
**Figure S6** The coding sequence of *TaDEP1* gene from bread wheat cultivar KN199.
**Figure S7** Sequence alignment of DEP1 homologs.
**Figure S8** Phylogenetic tree of DEP1 homologs.
**Figure S9** The mTaCOLD1 (M187K) protein interferes with the physical association between TaGα‐7A and TaDEP1.
**Table S1** Conserved amino acid sequences of COLD1 homologs in rice, diploid and tetraploid wheat
**Table S2** Primers used in this study
**Table S3** Primers used for qRT‐PCR in this study
**Table S4** Constructs used in this study

## References

[pbi13008-bib-0001] Assmann, S.M. (2005) G proteins Go green: a plant G protein signaling FAQ sheet. Science 310, 71–73. 16210528 10.1126/science.1118580

[pbi13008-bib-0002] Bommert, P. , Je, B.I. , Goldshmidt, A. and Jackson, D. (2013) The maize Galpha gene COMPACT PLANT2 functions in CLAVATA signalling to control shoot meristem size. Nature 502, 555–558. 24025774 10.1038/nature12583

[pbi13008-bib-0003] Botella, J.R. (2012) Can heterotrimeric G proteins help to feed the world? Trends Plant Sci. 17, 563–568. 22748359 10.1016/j.tplants.2012.06.002

[pbi13008-bib-0004] Chakravorty, D. , Trusov, Y. , Zhang, W. , Acharya, B.R. , Sheahan, M.B. , McCurdy, D.W. , Assmann, S.M. *et al* (2011) An atypical heterotrimeric G‐protein gamma‐subunit is involved in guard cell K(+)‐channel regulation and morphological development in *Arabidopsis thaliana* . Plant J. 67, 840–851. 21575088 10.1111/j.1365-313X.2011.04638.x

[pbi13008-bib-0005] Chen, J.G. (2008) Heterotrimeric G‐proteins in plant development. Front Biosci. 13, 3321–3333. 18508435 10.2741/2928

[pbi13008-bib-0006] Chen, H. , Zou, Y. , Shang, Y. , Lin, H. , Wang, Y. , Cai, R. , Tang, X. *et al* (2008) Firefly luciferase complementation imaging assay for protein‐protein interactions in plants. Plant Physiol. 146, 368–376. 18065554 10.1104/pp.107.111740 PMC2245818

[pbi13008-bib-0007] Deng, W. , Nickle, D.C. , Learn, G.H. , Maust, B. and Mullins, J.I. (2007) ViroBLAST: a stand‐alone BLAST web server for flexible queries of multiple databases and user's datasets. Bioinformatics 23, 2334–2336. 17586542 10.1093/bioinformatics/btm331

[pbi13008-bib-0008] Fujisawa, Y. , Kato, T. , Ohki, S. , Ishikawa, A. , Kitano, H. , Sasaki, T. , Asahi, T. *et al* (1999) Suppression of the heterotrimeric G protein causes abnormal morphology, including dwarfism, in rice. Proc. Natl Acad. Sci. USA 96, 7575–7580. 10377457 10.1073/pnas.96.13.7575 PMC22128

[pbi13008-bib-0009] Gilman, A.G. (1987) G proteins: transducers of receptor‐generated signals. Annu. Rev. Biochem. 56, 615–649. 3113327 10.1146/annurev.bi.56.070187.003151

[pbi13008-bib-0010] Huang, X. , Qian, Q. , Liu, Z. , Sun, H. , He, S. , Luo, D. , Xia, G. *et al* (2009) Natural variation at the DEP1 locus enhances grain yield in rice. Nat. Genet. 41, 494–497. 19305410 10.1038/ng.352

[pbi13008-bib-0011] International Wheat Genome Sequencing Consortium (IWGSC) . (2014) A chromosome‐based draft sequence of the hexaploid bread wheat (*Triticum aestivum*) genome. Science 345, 1251788. 25035500 10.1126/science.1251788

[pbi13008-bib-0012] Jia, J. , Zhao, S. , Kong, X. , Li, Y. , Zhao, G. , He, W. , Appels, R. *et al* (2013) Aegilops tauschii draft genome sequence reveals a gene repertoire for wheat adaptation. Nature 496, 91–95. 23535592 10.1038/nature12028

[pbi13008-bib-0013] Jones, A.M. (2002) G‐protein‐coupled signaling in *Arabidopsis* . Curr. Opin. Plant Biol. 5, 402–407. 12183178 10.1016/s1369-5266(02)00288-1

[pbi13008-bib-0014] Jones, A.M. and Assmann, S.M. (2004) Plants: the latest model system for G‐protein research. EMBO Rep. 5, 572–578. 15170476 10.1038/sj.embor.7400174 PMC1299082

[pbi13008-bib-0015] Jones, J.C. , Duffy, J.W. , Machius, M. , Temple, B.R. , Dohlman, H.G. and Jones, A.M. (2011) The crystal structure of a self‐activating G protein alpha subunit reveals its distinct mechanism of signal initiation. Sci. Signal. 4, ra8. 21304159 10.1126/scisignal.2001446 PMC3551277

[pbi13008-bib-0016] Lee, H.K. , Cho, S.K. , Son, O. , Xu, Z. , Hwang, I. and Kim, W.T. (2009) Drought stress‐induced Rma1H1, a RING membrane‐anchor E3 ubiquitin ligase homolog, regulates aquaporin levels via ubiquitination in transgenic *Arabidopsis* plants. Plant Cell 21, 622–641. 19234086 10.1105/tpc.108.061994 PMC2660634

[pbi13008-bib-0017] Li, Y. , Xiao, J. , Wu, J. , Duan, J. , Liu, Y. , Ye, X. , Zhang, X. *et al* (2012) A tandem segmental duplication (TSD) in green revolution gene Rht‐D1b region underlies plant height variation. New Phytol. 196, 282–291. 22849513 10.1111/j.1469-8137.2012.04243.x

[pbi13008-bib-0018] Ling, H.Q. , Zhao, S. , Liu, D. , Wang, J. , Sun, H. , Zhang, C. , Fan, H. *et al* (2013) Draft genome of the wheat A‐genome progenitor *Triticum urartu* . Nature 496, 87–90. 23535596 10.1038/nature11997

[pbi13008-bib-0019] Liu, J. , Cheng, X. , Liu, P. and Sun, J. (2017) miR156‐targeted SBP‐box transcription factors interact with DWARF53 to regulate TEOSINTE BRANCHED1 and BARREN STALK1 expression in bread wheat. Plant Physiol. 174, 1931–1948. 28526703 10.1104/pp.17.00445 PMC5490914

[pbi13008-bib-0020] Lu, Q. , Tang, X. , Tian, G. , Wang, F. , Liu, K. , Nguyen, V. , Kohalmi, S.E. *et al* (2010) *Arabidopsis* homolog of the yeast TREX‐2 mRNA export complex: components and anchoring nucleoporin. Plant J. 61, 259–270. 19843313 10.1111/j.1365-313X.2009.04048.x

[pbi13008-bib-0021] Ma, H. , Yanofsky, M.F. and Meyerowitz, E.M. (1990) Molecular cloning and characterization of GPA1, a G protein alpha subunit gene from *Arabidopsis thaliana* . Proc. Natl Acad. Sci. USA 87, 3821–3825. 2111018 10.1073/pnas.87.10.3821 PMC53995

[pbi13008-bib-0022] Ma, Y. , Dai, X. , Xu, Y. , Luo, W. , Zheng, X. , Zeng, D. , Pan, Y. *et al* (2015) COLD1 confers chilling tolerance in rice. Cell 160, 1209–1221. 25728666 10.1016/j.cell.2015.01.046

[pbi13008-bib-0023] Mason, M.G. and Botella, J.R. (2000) Completing the heterotrimer: isolation and characterization of an *Arabidopsis thaliana* G protein gamma‐subunit cDNA. Proc. Natl Acad. Sci. USA 97, 14784–14788. 11121078 10.1073/pnas.97.26.14784 PMC18996

[pbi13008-bib-0024] Mason, M.G. and Botella, J.R. (2001) Isolation of a novel G‐protein gamma‐subunit from *Arabidopsis thaliana* and its interaction with Gbeta. Biochim. Biophys. Acta 1520, 147–153. 11513956 10.1016/s0167-4781(01)00262-7

[pbi13008-bib-0025] Nakagawa, T. , Kurose, T. , Hino, T. , Tanaka, K. , Kawamukai, M. , Niwa, Y. , Toyooka, K. *et al* (2007) Development of series of gateway binary vectors, pGWBs, for realizing efficient construction of fusion genes for plant transformation. J. Biosci. Bioeng. 104, 34–41. 17697981 10.1263/jbb.104.34

[pbi13008-bib-0026] Peng, J. , Richards, D.E. , Hartley, N.M. , Murphy, G.P. , Devos, K.M. , Flintham, J.E. , Beales, J. *et al* (1999) ‘Green revolution’ genes encode mutant gibberellin response modulators. Nature 400, 256–261. 10421366 10.1038/22307

[pbi13008-bib-0027] Perfus‐Barbeoch, L. , Jones, A.M. and Assmann, S.M. (2004) Plant heterotrimeric G protein function: insights from *Arabidopsis* and rice mutants. Curr. Opin. Plant Biol. 7, 719–731. 15491922 10.1016/j.pbi.2004.09.013

[pbi13008-bib-0028] Qin, L. , Zhao, J. , Li, T. , Hou, J. , Zhang, X. and Hao, C. (2017) TaGW2, a good reflection of wheat polyploidization and evolution. Front. Plant Sci. 8, 318. 28326096 10.3389/fpls.2017.00318 PMC5339256

[pbi13008-bib-0029] Shan, Q. , Wang, Y. , Li, J. , Zhang, Y. , Chen, K. , Liang, Z. , Zhang, K. *et al* (2013) Targeted genome modification of crop plants using a CRISPR‐Cas system. Nat. Biotechnol. 31, 686–688. 23929338 10.1038/nbt.2650

[pbi13008-bib-0030] Sun, J. , Qi, L. , Li, Y. , Zhai, Q. and Li, C. (2013) PIF4 and PIF5 transcription factors link blue light and auxin to regulate the phototropic response in *Arabidopsis* . Plant Cell 25, 2102–2114. 23757399 10.1105/tpc.113.112417 PMC3723615

[pbi13008-bib-0031] Sun, H. , Qian, Q. , Wu, K. , Luo, J. , Wang, S. , Zhang, C. , Ma, Y. *et al* (2014) Heterotrimeric G proteins regulate nitrogen‐use efficiency in rice. Nat. Genet. 46, 652–656. 24777451 10.1038/ng.2958

[pbi13008-bib-0032] Urano, D. and Jones, A.M. (2014) Heterotrimeric G protein‐coupled signaling in plants. Annu. Rev. Plant Biol. 65, 365–384. 24313842 10.1146/annurev-arplant-050213-040133 PMC4861148

[pbi13008-bib-0033] Urano, D. , Chen, J.G. , Botella, J.R. and Jones, A.M. (2013) Heterotrimeric G protein signalling in the plant kingdom. Open Biol. 3, 120186. 23536550 10.1098/rsob.120186 PMC3718340

[pbi13008-bib-0034] Urano, D. , Miura, K. , Wu, Q. , Iwasaki, Y. , Jackson, D. and Jones, A.M. (2016) Plant morphology of heterotrimeric G protein mutants. Plant Cell Physiol. 57, 437–445. 26755691 10.1093/pcp/pcw002 PMC4900173

[pbi13008-bib-0035] Van De Velde, K. , Chandler, P.M. , Van Der Straeten, D. and Rohde, A. (2017) Differential coupling of gibberellin responses by Rht‐B1c suppressor alleles and Rht‐B1b in wheat highlights a unique role for the DELLA N‐terminus in dormancy. J. Exp. Bot. 68, 443–455. 28073950 10.1093/jxb/erw471 PMC5853533

[pbi13008-bib-0036] Weiss, C.A. , Garnaat, C.W. , Mukai, K. , Hu, Y. and Ma, H. (1994) Isolation of cDNAs encoding guanine nucleotide‐binding protein beta‐subunit homologues from maize (ZGB1) and *Arabidopsis* (AGB1). Proc. Natl Acad. Sci. USA 91, 9554–9558. 7937804 10.1073/pnas.91.20.9554 PMC44851

[pbi13008-bib-0037] Wendt, T. , Holme, I. , Dockter, C. , Preuss, A. , Thomas, W. , Druka, A. , Waugh, R. *et al* (2016) HvDep1 is a positive regulator of culm elongation and grain size in barley and impacts yield in an environment‐dependent manner. PLoS ONE 11, e0168924. 28005988 10.1371/journal.pone.0168924 PMC5179111

[pbi13008-bib-0038] Wu, J. , Kong, X. , Wan, J. , Liu, X. , Zhang, X. , Guo, X. , Zhou, R. *et al* (2011) Dominant and pleiotropic effects of a GAI gene in wheat results from a lack of interaction between DELLA and GID1. Plant Physiol. 157, 2120–2130. 22010107 10.1104/pp.111.185272 PMC3327208

[pbi13008-bib-0039] Yoo, S.D. , Cho, Y.H. and Sheen, J. (2007) *Arabidopsis* mesophyll protoplasts: a versatile cell system for transient gene expression analysis. Nat. Protoc. 2, 1565–1572. 17585298 10.1038/nprot.2007.199

[pbi13008-bib-0040] Zhang, Y. , Liang, Z. , Zong, Y. , Wang, Y. , Liu, J. , Chen, K. , Qiu, J.L. *et al* (2016) Efficient and transgene‐free genome editing in wheat through transient expression of CRISPR/Cas9 DNA or RNA. Nat. Commun. 7, 12617. 27558837 10.1038/ncomms12617 PMC5007326

