# Supplementary material for: TaCOLD1 defines a new regulator of plant height in bread wheat
Source: Plant Biotechnol J. 2018 Sep 24;17(3):687–99. doi: 10.1111/pbi.13008 (PMC6381784; doi:10.1111/pbi.13008)
Supplement: Supplementary file 1 — Figure S1 The coding sequences of TaCOLD1‐2A/2B/2D genes from bread wheat cultivar KN199. Figure S2 Topology prediction for TaCOLD1‐2A/2B proteins using a transmembrane domain hidden Markov model (TMHMM version 2.0). Figure S3 The coding sequence of Rht‐B1b gene from bread wheat cultivar KN199. Figure S4 Phenotypes of WT KN199 and pUbi:mTaCOLD1 transgenic wheat lines grown in the field at vegetative stage. Figure S5 The coding sequences of TaGα‐7A/1B/7D genes from bread wheat cultivar KN199. Figure S6 The coding sequence of TaDEP1 gene from bread wheat cultivar KN199. Figure S7 Sequence alignment of DEP1 homologs. Figure S8 Phylogenetic tree of DEP1 homologs. Figure S9 The mTaCOLD1 (M187K) protein interferes with the physical association between TaGα‐7A and TaDEP1. Table S1 Conserved amino acid sequences of COLD1 homologs in rice, diploid and tetraploid wheat Table S2 Primers used in this study Table S3 Primers used for qRT‐PCR in this study Table S4 Constructs used in this study [file PBI-17-687-s001.pdf]

Supplemental Data

Figure S1

TaCOLD1-2A

5’-
ATGGGGTGGGGCACGGTGGTCTACGAGGGCACGGTGGTGGGGGCGTCGCTGGTGGGGCTGGGGTGGGC
GGGGCTGTGGTTCCTCAACCGGCGGCTGTACAAGGAGTACGAGGAGCGGCGGGCGCTGGTGCAGATCCT
CTTCGGCCTCGTCTTCGCCTTCTCCTGCAACCTCTTCCAGCTCGTCCTCTTCGAGATCCTCCCCGTCCTCTC
CAAGCACGCGCGCTTCCTCAACTGGCACCTCGACCTCTTCTGCCTCATCCTCCTCCTCGTCTTCCTCCTCC
CCTACTACCACTGCTACCTGCTGCTCCGCAACTCAGGGGTGAGAAGGCAGCGGGCCTGCCTCGCCGCGGC
CCTCTTCCTCACCATCTTCCTCTACGGGTTCTGGCGCATGGGGATTCACTTCCCTATGCCTTCCCCAGAGAA
AGGTTTCTTTACGATGCCGCAGTTGGTTAGTAGGATTGGCGTGATTGGAGTGACTGTCATGGCTGTTCTTTC
TGGTTTTGGTGCGGTCAATCTGCCATACAGCTACCTGTCTCTCTTTATCAGGGAAATTGACGAAATGGACAT
CAAAGCTTTGGAACGGCAGCTGATGCAATCCATGGAGTCATCTATTGCTAAGAAAAAGAAAATTATTCTGTC
CAAAATGGAGATGGAGAGGATCCAAGGATCAGAGGAGAAGCTTAAAGCCAGATCATTTCTGAAGCGTATAG
TTGGAACAGTTGTTCGATCTGTGCAAGAAGATCAAACTGAGCAGGATATCAAAAACCTGGAAGCAGAGGTC
CAGGGGTTGGAAGAGCTTTCCAAACAGCTATTCCTTGAGATATATGAACTACGTCAAGCCAAGATAGCTGCT
GCTTATTCCCGAACCTGGAGAGGACATTTTCAGAATCTGCTAGGATATGCCTTGTCAGTATATTGTGTTTATA
AGATGCTCAAGGCCTTGCAGAGCGTAGTCTTTAAAGCGGCTGGCTCTGTTGATCCGGTAACCATGACGATT
ACCATTTTCTTGAGGCATTTTGATATCGGTATTGATGTCACGCTATTATCACAGTACATATCTTTGTTGTTCAT
CGGGATGTTGGTTGGCATATCTATTCGAGGTTTCTTGGCAAATGTTATGAAGTTCTTCTTTGCTTTTTCTAGA
GTTGGGACCGGCTCAACAACCAATGTCGTCCTTTTCTTATCTGAGATCATGGGCATGTATTTCATATCTTCTA
TTCTTCTGATAAGGAAAAGCCTGGCAAATGAGTACAGGGTGATCATCACAGATGTTTTGGGCGGTGACATTC
AGTTTGACTTCTACCACCGTTGGTTTGATGCTATATTTGTGGCGAGCGCGTTCTTGTCCTTGCTTTTGATTTC
AGCTCAGTACACCTCCCGGCAAGCAGACAAGCATCCAATTGATTGA-3’

TaCOLD1-2B

5’-
ATGGGGTGGGGCGTGGTGGTCTACGAGGGCACGGTGGTGGGGGCGTCGCTGGTGGGGCTGGGGTGGGC
GGGGCTGTGGTTCCTCAACCGGCGGCTGTACAAGGAGTACGAGGAGCGGCGGGCGCTGGTGCAGATCCT
CTTCGGCCTCGTCTTCGCCTTCTCCTGCAACCTCTTCCAGCTCGTCCTCTTCGAGATCCTCCCCGTCCTCTC
CAAGCACGCGCGCTTCCTCAACTGGCACCTCGACCTCTTCTGCCTCATCCTCCTCCTCGTCTTCGTCCTCC
CCTATTACCACTGCTACCTGCTGCTCCGCAACTCAGGTGTGAGGAGGCAGCGGGCCTGCCTCGCCGCGGC
CCTCTTCCTGACCATCTTCCTCTACGGGTTCTGGCGCATGGGGATTCACTTCCCCATGCCTTCTCCGGAGA
AAGGTTTCTTTACGATGCCGCAGTTGGTTAGTAGGATTGGCGTGATTGGAGTGACTGTCATGGCTGTCCTTT
CTGGTTTTGGTGCGGTCAATCTGCCATACAGCTACCTGTCTCTCTTTATCAGGGAAATTGACGAAATGGACA
TCAAAGCTTTGGAACGGCAGCTGATGCAATCCATGGAGTCATCTATTGCTAAGAAAAAGAAAATTATTCTGT
CCAAAATGGAGATGGAGAGGATCCAAGGATCAGAGGAGAAGCTTAAAGCCAGATCATTTCTGAAGCGTATA
GTTGGAACAGTGGTTCGATCTGTGCAAGAAGATCAAACTGAGCAGGATATCAAAAACTTGGAAGCAGAGGT
CCAGGGATTGGAAGAGCTTTCGAAACAGCTATTCCTTGAGATATATGAACTACGTCAAGCTAAGATAGCTGC
TGCTTATTCCCGAACCTGGAGAGGACATTTTCAGAATCTGCTAGGATATGCCTTGTCAGTGTATTGTGTTTAT
AAGATGCTCAAGGCCTTGCAGAGTGTAGTCTTTAAAGCGGCTGGCTCTGTTGATCCGGTAACCATGACGAT
TACCATTTTCTTGAGGCATTTTGATATTGGTATCGATGTCACGCTATTATCACAGTACATATCTTTGTTGTTCA
TCGGGATGTTGGTTGGCATATCTATTCGAGGTTTCTTGGCAAATGTTATGAAGTTCTTCTTTGCTTTTTCTAG
AGTTGGGACCGGCTCAACAACAAATGTCGTCCTTTTCTTATCTGAGATCATGGGCATGTATTTCATATCTTCT
ATTCTTCTGATAAGGAAAAGCCTGGCAAATGAGTACAGGGTGATCATCACAGATGTTTTGGGCGGTGACATT
CAGTTTGACTTCTACCACCGCTGGTTTGATGCCATATTCGTGGCGAGCGCGTTCTTGTCCTTGCTTTTGATT
TCAGCCCAGTACACCTCCCGGCAAGCAGACAAGCATCCAATTGATTGA-3’

Figure S1 continued

TaCOLD1-2D

5’-
ATGGGGTGGGGCGTGGTGGTCTACGAGGGCACGGTGGTGGGGGCGTCGCTGGTGGGGCTGGGGTGGGC
GGGGCTGTGGTTCCTCAACCGGCGGCTGTACAAGGAGTACGAGGAGCGGCGGGCGCTGGTGCAGATCCT
CTTCGGCCTCGTCTTCGCCTTCTCCTGCAACCTCTTCCAGCTCGTCCTCTTCGAGATCCTCCCCGTCCTCTC
CAAGCACGCGCGCTTCCTCAACTGGCACCTCGACCTCTTCTGCCTCATCCTCCTCCTCGTCTTCCTCCTCC
CCTACTACCACTGCTACCTGCTGCTCCGCAACTCAGGGGTGAGAAGGCAGCGGGCCTGCCTCGCCGCGGC
CCTCTTCCTCACCATCTTCCTCTACGGGTTCTGGCGCATGGGGATTCACTTCCCCATGCCTTCCCCAGAGA
AAGGTTTCTTTACGATGCCGCAGTTGGTTAGTAGGATTGGCGTGATTGGAGTGACTGTCATGGCTGTTCTTT
CTGGTTTTGGTGCGGTCAATCTGCCATACAGCTACCTGTCTCTCTTTATCAGGGAAATTGACGAAATGGACA
TCAAAGCTTTGGAGCGGCAGCTGATGCAATCCATGGAGTCATCTATTGCTAAGAAAAAGAAAATTATTCTGT
CCAAAATGGAGATGGAGAGGATCCAAGGATCAGAGGAGAAGCTTAAAGCCAGATCATTTCTGAAGCGTATA
GTTGGAACAGTTGTTCGATCTGTGCAAGAAGATCAAACTGAGCAGGATATCAAAAACCTGGAAGCAGAGGT
CCAGGGGTTGGAAGAGCTTTCCAAACAGCTATTCCTTGAGATATATGAACTACGTCAAGCCAAGATAGCTGC
TGCTTATTCCCGAACCTGGAGAGGACATTTTCAGAATCTGCTAGGATATGCCTTGTCAGTATATTGTGTTTAT
AAGATGCTCAAGGCCTTGCAGAGCGTAGTCTTTAAAGCGGCTGGCTCTGTTGATCCGGTAACCATGACGAT
TACCATTTTCTTGAGGCATTTTGATATCGGTATTGATGTCACGCTATTATCACAGTACATATCTTTGTTGTTCA
TCGGGATGTTGGTTGGCATATCTATTCGAGGTTTCTTGGCAAATGTTATGAAGTTCTTCTTTGCTTTTTCTAG
AGTTGGGACCGGCTCAACAACCAATGTCGTCCTTTTCTTATCTGAGATCATGGGCATGTATTTCATATCTTCT
ATTCTTCTGATAAGGAAAAGCCTGGCAAATGAGTACAGGGTGATCATCACAGATGTTTTGGGCGGTGACATT
CAGTTTGACTTCTACCACCGTTGGTTTGATGCTATATTTGTGGCGAGCGCGTTCTTGTCCTTGCTTTGA-3’

Figure S1. The coding sequences of *TaCOLD1-2A/2B/2D* genes from bread wheat cultivar KN199.

Figure S2

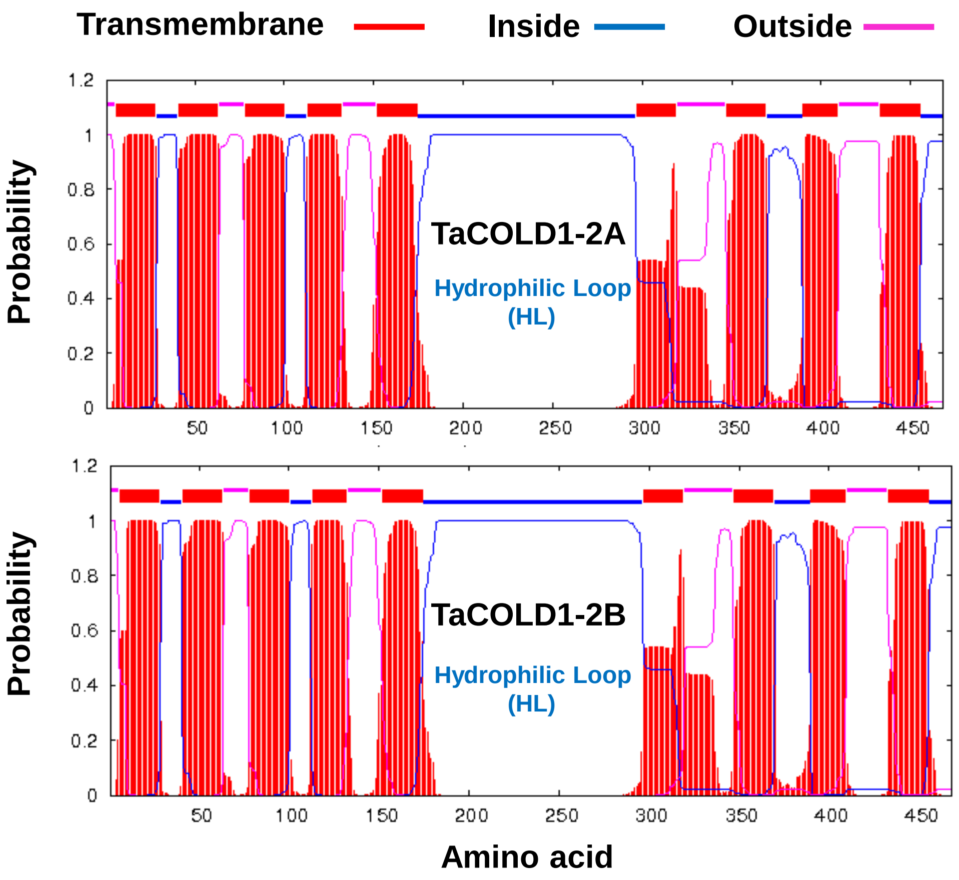

Figure S2. Topology prediction for TaCOLD1-2A/2B proteins using a transmembrane domain hidden Markov model (TMHMM version 2.0).

Figure S3

Rht-B1b

5’-
ATGAAGCGCGAGTACCAGGACGCCGGAGGGAGCGGCGGTGGCGGGGGAGGCATGGGCTCGTCCGAGGA
CAAGATGATGGTGTCGGGGTCGGCGGCGGCGGGGGAGGGGGAGGAGGTGGACGAGCTGCTGGCGGCGC
TCGGGTACAAGGTGCGGGCGTCCGACATGGCGGACGTGGCGCAGAAGCTGGAGTAGCTGGAGATGGCCA
TGGGGATGGGCGGCGTGGGCGCCGGCGCCGCGCCCGACGACAGCTTCGCCACCCACCTCGCCACGGAC
ACCGTGCACTACAACCCCACCGACCTCTCCTCCTGGGTCGAGAGCATGCTGTCGGAGCTCAACGCGCCGC
CGCCGCCCCTCCCGCCCGCCCCGCAGCTCAACGCCTCCACCTCCTCCACCGTCACCGGCGGCGGGTACT
TCGATCTCCCGCCCTCCGTCGACTCCTCCTGCAGCACCTACGCGCTGCGGCCGATCCCGTCCCCGGCCGT
CGCGCCGGCCGACCTCTCCGCCGACTCCGTCGTGCGGGATCCCAAGCGGATGCGCACTGGCGGCAGCAG
CACCTCGTCGTCATCCTCATCGTCCTCTCTCGGCGGTGGCGGCGCCAGGAGCTCTGTGGTGGAGGCTGCC
CCGCCGGTGGCCGCCGCGGCCGGTGCGCCCGCGCTGCCGGTCGTCGTGGTCGACACGCAGGAGGCCGG
GATTCGGCTGGTGCACGCGCTGCTGGCGTGCGCAGAGGCCGTGCAGCAGGAGAACTTCTCTGCCGCGGA
GGCGCTGGTGAAGCAGATACCCTTGCTGGCCGCGTCCCAGGGCGGCGCCATGCGCAAGGTCGCCGCCTA
CTTCGGCGAGGCCCTCGCCCGCCGCGTCTTCCGCTTCCGCCCGCAGCCGGACAGCTCCCTCCTCGACGC
CGCCTTCGCCGACCTCCTCCACGCGCACTTCTACGAGTCCTGCCCCTACCTCAAGTTCGCCCACTTCACCG
CCAACCAGGCCATCCTGGAGGCGTTCGCCGGCTGCCGCCGCGTGCACGTCGTCGACTTCGGCATCAAGC
AGGGGATGCAGTGGCCCGCCCTTCTCCAGGCCCTGGCGCTCCGTCCCGGCGGCCCTCCCTCGTTCCGCC
TCACCGGCGTCGGCCCCCCGCAGCCGGACGAGACCGACGCCTTGCAGCAGGTGGGCTGGAAGCTCGCCC
AGTTCGCGCACACCATCCGCGTCGACTTCCAGTACCGCGGCCTCGTCGCCGCCACGCTCGCGGACCTGGA
GCCGTTCATGCTGCAGCCGGAGGGCGAGGAGGACCCGAACGAGGAGCCCGAGGTAATCGCCGTCAACTC
GGTCTTCGAGATGCACCGGCTGCTCGCGCAGCCCGGCGCCCTGGAGAAGGTCCTGGGCACCGTGCGCGC
CGTGCGGCCGAGGATCGTCACCGTGGTGGAGCAGGAGGCGAACCACAACTCCGGCACATTCCTGGACCG
CTTCACCGAGTCCCTGCACTACTACTCCACCATGTTCGATTCTCTGGAGGGCGGCAGCTCCGGCGGCCCAT
CCGAAGTCTCATCTGGGGCGGCTGCTGCTCCTGCCGCCGCCGGCACGGACCAGGTCATGTCCGAGGTGT
ACCTCGGCCGGCAGATCTGCAACGTGGTGGCCTGCGAGGGGGCGGAGCGCACAGAGCGGCACGAGACC
CTGGGGCAGTGGCGGAACCGCCTCGGCAACGCCGGGTTCGAGACCGTCCACCTGGGCTCCAATGCCTAC
AAGCAGGCGAGCACGCTGCTGGCGCTCTTCGCAGGCGGCGACGGGTACAAGGTGGAGGAGAAGGAGGGC
TGCCTGACGCTGGGGTGGCACACGCGCCCGCTGATCGCCACCTCCGCATGGCGCCTGGCCGCGCCGTGA
-3’

Figure S3. The coding sequence of *Rht-B1b* gene from bread wheat cultivar KN199.

Figure S4

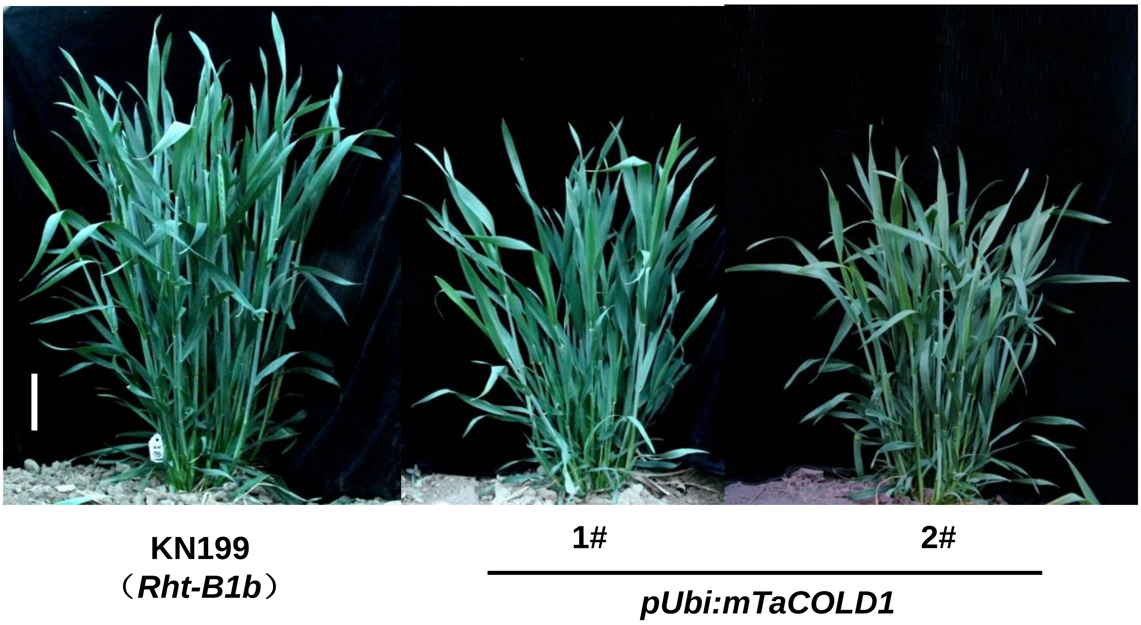

Figure S4. Phenotypes of WT KN199 and *pUbi:mTaCOLD1* transgenic wheat lines grown in the field at vegetative stage. Scale bar, 10 cm.

Figure S5

TaGα-7A

5’-
ATGTCCATGCTCGCGTGTGCGCTTCAAACCATGGGCTCATCCTGCAGCAGACCTCACTCAGTAAATGAGGC
AGACGCAGCTGACAACACAAGATCTGCAGACATCGACCGCCGCATTCTGCACGAGACAAAGGCGGACCAG
CACATCCACAAGCTCTTGCTTCTTGGTGCCGGAGAATCAGGAAAGTCCACGATATTTAAACAGATCAAGCTT
CTTTTCCGAACCGGCTTCGACGAGGCAGAACTCAAGGGCTATACGCCCGTCATCCATGCCAACGTGTTCC
AGACAATCAAAATACTATATGATGGAGCTAAAGAGCTTGCCCAAGTGGAATCCGAGTCTTCAAAATATGTGA
TGTTACCCGATAATCAGGAGATTGGAGAAAAACTATCAGAAATCGGAGGCAGGTTGGATTACCCTTCGCTT
AACAAAGAACTCGTACAGGATGTGAGAAAATTATGGGAAGATCAAGCCATTCAGGAAACTTACTCGTGTGG
AAGTGTGCTGCAAGTTCCTGACTGTGCACACTACTTCATGGACAATTTGGACCGATTAGCTGAAGCAGATT
ACGTACCAACAAAGGAGGATGTGCTCCATGCAAGAGTGCGGACAAATGGGGTTGTAGAAATTCAGTTTAGC
CCCCTTGGAGAGAGCAAAAGGGGCGGAGAGGTGTACAGGCTGTACGACGTAGGGGGTCAAAGGAATGAG
AGGAGGAAGTGGATTCATCTCTTTGAAGGTGTTGATGCAGTTATCTTTTGTGCTGCCATTAGCGAGTACGAT
CAGTTGTTATTTGAGGATGAGACGCAGAACAGGATGATGGAGACCAAGGAGCTGTTCGACTGGGTGTTAAA
GCAAACATGTTTTGAGAAAACATCCTTCATGCTGTTCCTCAACAAATTTGACATATTCGAGAGGAAAATACA
AAAGGTTCCTTTGACCGTGTGCGAGTGGTTTAAGGACTATGAGCCAATCGCGCCTGGCAAACAGGATGTG
GAGCATGCCTACGAGTTCGTGAAGAAGAAGTTTGAGGAGGTCTACTTCCAGAGCAGCAAGCCCGAGCGTG
TCGACCGGGTGTTCAAGATCTACAGAACGACGGCGCTGGACCAGAAGCTTGTGAAGAAGACGTTCAAGCT
GATGGACGAGAGCATGAGACGCTCCCGGGAAGGAACGGGGACGTGA-3’

TaGα-1B

5’-
ATGTCCATGCTCGCGTGCGCGCTTCAAACCATGGGCTCCTCCTGCAGCAGACCTCACTCCGTAAACGAGGC
CGAGGCAGCCGACAACACAAGATCTGCAGACATCGACCGGCGGATTCTGCAGGAGACAAAGGCGGATCAG
CACGTCCACAAGCTCTTGCTTCTCGGTGCTGGAGAATCAGGAAAGTCCACGATATTTAAGCAGATTAAGCTTC
TTTTTCGAACCGGCTTCGACGAGGCAGAACTCAAGGGCTATATGCCGGTCATCCATGCCAACGTGTTCCAGA
CAATCAAAATACTGTATGATGGAGCTAAAGAGCTTGCCCAACTGGAAACTGAGTCTTCAAAACATGTTATATC
CCCGGATAATCAGGAGATTGGAGAAAAACTATCAGAAATCGGAGGCAGGTTGGATTACCCACTCCTTAACAA
AGAACTCGTACAGGATGTAAGAAAATTATGGGAAGATTCAGCCATTCAGGAAACTTACTCGTGTGGAAGTGTG
CTGCAAGTTCCTGATTGTGCACACTACTTCATGGAGAATCTGGACCGATTAGCTGAACCAGATTATATACCAA
CAAAGGAGGATGTGCTCCATGCCAGAGTACGGACAAATGGGGTTGTGGAAATTCAATTTAGCCCCCTTGGAG
AGAGTAAAAGAGGCGGAGAGGTATACAGGTTGTACGATGTAGGAGGTCAAAGGAATGAGAGGAGGAAGTGG
ATTCATCTTTTTGAAGGCGTCGATGCCGTCATCTTTTGCGCTGCCATTAGCGAGTATGATCAGCTGTTGTTTG
AGGACGAGACACAGAACAGAATGATGGAGACGAAGGAACTGTTCGACTGGGTACTAAAGCAAAGATGTTTTG
AGAAAACATCGTTCATGCTGTTCCTCAACAAATTCGACATATTTGAGAGGAAAATACAAAAGGTTCCTTTGAC
CGTGTGCGAGTGGTTTAAAGATTATGAGCCGATCGCGCCTGGCAAACAGGATGTGGAACATGCCTATGAGTT
TGTGAAGAAGAAATTTGAGGAGGTCTACTTCCAGAGCAGCAAGCCGGACCGTGTGGACCGGGTGTTCAAGA
TCTACGGGTGTTCAAGATCTACAGAACGACGGCGCTGGACCAGAAACTTGTAA-3’

TaGα-7D

5’-
ATGCTCGCGTGTGCGCTTCAAACCATGGGCTCATCCTGCAGCAGACCTCACTCAGTAAATGAGGCAGACGC
AGCTGACAACACAAGATCTGCAGACATCGACCGGCGGATTCTTCAGGAGACAAAGGCGGACCAGCACATCC
ACAAGCTCTTGCTTCTTGGTGCCGGAGAATCAGGAAAGTCCACGATATTTAAACAGATCAAGCTTCTTTTCCG
AACCGGCTTCGACGAGGCAGAACTCAAGGGCTATACGCCCGTCATCCATGCCAACGTGTTCCAGACAATCAA
AATACTATACGATGGAGCTAAAGAGCTTGCCCAAGTGGAACCCGAGTCTTCAAAATATGTGATATTACCCGAT
AATCAGGAGATTGGAGAAAAACTATCAGAAATCGGAGGCAGGTTGGATTACCCGTTGCTTAACAAAGAACTC
GTACAGGATGTAAGAAAATTATGGGAAGATCAAGCCATTCAGGAAACTTACTCGTGTGGAAGTGTGCTGCAA
GTTCCTGACTGTGCACACTACTTCATGGACAATTTGGACCGATTAGCTGAAGCAGATTACGTACCAACAAAGG
AGGATGTGCTCCATGCAAGAGTGCGGACAAATGGGGTTGTAGAAATTCAATTTAGCCCCCTTGGAGAGAGCA
AAAGGGGCGGAGAGGTGTACAGGCTGTACGACGTAGGGGGTCAAAGGAATGAGAGAAGGAAGTGGATTCAT
CTCTTTGAAGGTGTTGATGCAGTTATCTTTTGTGCTGCCATTAGCGAGTACGATCAGTTGTTATTTGAGGACG
AGACGCAGAACAGGATGATGGAGACCAAGGAGCTGTTCGACTGGGTATTAAAGCAGAGATGTTTTGAGAAAA
CATCCTTCATGTTGTTCCTCAACAAATTTGACATATTCGAGAGGAAAATACAAAAGGTTCCTTTGACCGTGTGC
GAGTGGTTTAAGGACTATGAGCCAATCGCGCCTGGCAAACAGGATGTGGAGCATGCCTATGAGTTCGTGAA
GAAGAAGTTTGAGGAGGTCTACTTCCAGAGCAGCAAGCCCGAGCGTGTCGACCGGGTGTTCAAGATCTACA
GAACGACAGCGCTGGACCAGAAACTTGTAAAGAAGACGTTCAAGCTGATGGACGAGAGCATGAGACGCTCC
CGGGAAGGAACGGGGACGTGA-3’

Figure S5. The coding sequences of *TaGα-7A/1B/7D* genes from bread wheat cultivar KN199.

Figure S6

TaDEP1

5'-
ATGGGGGAGGGCGCGGTGGTGGTGCTGGAGGCGCCCAAGCCCAGGTCGCCGCCGAGGTACCCGGACAT
GTGCGGTCGCCGGCGCCTGCAGCTGGAGGTGCAGATCCTTGACCGCGAGCTCACGTTCCTCAAGGACGA
GCTACATTTACTTGAAGGGGCTCAACCAGTCTCACGTTCTGGTTGCTTGAAAGAGGTAAACGAGTTTGTTG
GTACAAAACAAGACCCGCTAATACCAATTAACAAAAGGAAGCACCGGTCCTGCCGTCTTTATTGGTGGATC
AGATCGAAACTGTGCATATGTGCTTCATGGCTGTGCTGCTCCTGCCAATGCCTACCAACCTGCAAAAGACC
AAGGTGCTTCGACTGTTCATGCTGCGAGCCAAACTGCTCGTGCTGCAGCCTGAACTGCTGCAGCTGCTTC
AGTATCCCTTCGTGCTGCAAACCAAGCTGTGGCTGCTTTGAGTGCTGCAGCTGCAGCAAACCACAGTGCT
GCAGCAGCGGCTGTAACCCTTGCGGCGAGTGCAAGCCGGAGTGCGGCTCGTGTTCCGGCGGCGGCTGC
TGCGGCGAGCAGTGCTGCTCCTGCCCTCGATGCACAGGCTGCTTCAGCTGCTTCAAGGTCCCCAAATGCT
CGTGCGCGCAGTGCTTCAACTGCCAGTCGTCGTGCTGCAAGGGGCAGCCGTCGTGCTTCAGGTGCCAGT
CGTCGTGCTGCGACAAGGGAGGCTGCTGCAGCGGCGGGTCGTGCCTGAGCTGCCCGAAGCCGTCGTGC
CCGGAGTGCTCCTGCGGGTGCGTGTGGTCGTGCAAAAACTGTACAGACGGATGCCGATGCGCCCGGTGC
TGTGCTGGCGGGTGCCTGTGTTAA-3'

Figure S6. The coding sequence of *TaDEP1* gene from bread wheat cultivar KN199.

Figure S7

```
TaDEP1 : MGEGAVVVLEAPKPRSPPRYPDMCGRRRLQLEVQILDRELTFLKDELHLLEGAQPVSRSGCLKEVNEFVGTKQDPLIPIN :  80
HvDEP1 : MGEGAVVVLEPPKPRSPPRYPDMCGRRRLQLEVQILDRELTFLKDELHLLEGAQPVSRSACLKEVNEFVGTKQDPLIPIN :  80
OsDEP1 : MGE-EAVVMEAPRPKSPPRYPDLCGRRRMQLEVQILSREITFLKDELHFLEGAQPVSRSGCIKEINEFVGTKHDPLIPTK :  79

TaDEP1 : KRKHRSCRLYWWIRSKLCICASWLCCSCQCLPTCKRPRCFDCSC--------CEPNCS------CCSLNCCSCFS----- : 141
HvDEP1 : KRKHRSCRLYWWIRSKLCVCASWLCCSCQCLPTCKRPSCLDCSC--------CEPNCS------CCSPNCCSCFK----- : 141
OsDEP1 : RRRHRSCRLFRWIGSKLCICISCLCYCCKCSPKCKRPRCLNCSCSSCCDEPCCKPNCSACCAGSCCSPDCCSCCKPNCSC : 159

TaDEP1 : --IPSCCKP--SCGCFEC--------------------------------------CSCSKPQCCSS------------- : 166
HvDEP1 : --IPSCCKP--SCGCFGC--------------------------------------CSCSKPQCCSG------------- : 166
OsDEP1 : CKTPSCCKPNCSCSCPSCSSCCDTSCCKPSCTCFNIFSCFKSLYSCFKIPSCFKSQCNCSSPNCCTCTLPSCSCKGCACP : 239

TaDEP1 : --------------------------------GCNPCG-----ECKPECGSCSG---------GGCCG------------ : 188
HvDEP1 : --------------------------------GCNPCG-----ECKPECGSCSA---------GGCCGDCKPSCSCCGEQ : 200
OsDEP1 : SCGCNGCGCPSCGCNGCGCPSCGCNGCGLPSCGCNGCGSCSCAQCKPDCGSCSTNCCSCKPSCNGCCG----EQCCRCAD : 315

TaDEP1 : EQCCSCPRCTGCFSCFKVPKCSCA-------------QCFNCQSSCCKGQPSCFRCQSSCCDKGGCCSGGSCLSCPKPSC : 255
HvDEP1 : CQCCSCPRCTG--GCFKLPKCSCA-------------QCFNCQSSCCKGQPSCFRCQSSCCDKGGCCSSGSCLSCPKPSC : 265
OsDEP1 : CFSCSCPRCSS---CFNIFKCSCAGCCSSLCKCPCTTQCFSCQSSCCKRQPSCCKCQSSCCEGQPSCCEGHCCSLPKPSC : 392

TaDEP1 : PECSCGCVWSCKNCTDGCRCARC----CAGGCLC : 285
HvDEP1 : PECSCGCVWSCKNCTDGCRCARC----CASGCLC : 295
OsDEP1 : PECSCGCVWSCKNCTEGCRCPRCRNPCCLSGCLC : 426
```

Figure S7. Sequence alignment of DEP1 homologs. White or gray background represents the identity of amino acid sequences. Os, *Oryza sativa*; Hv, *Hordeum vulgare*; Ta, *Triticum aestivum*.

Figure S8

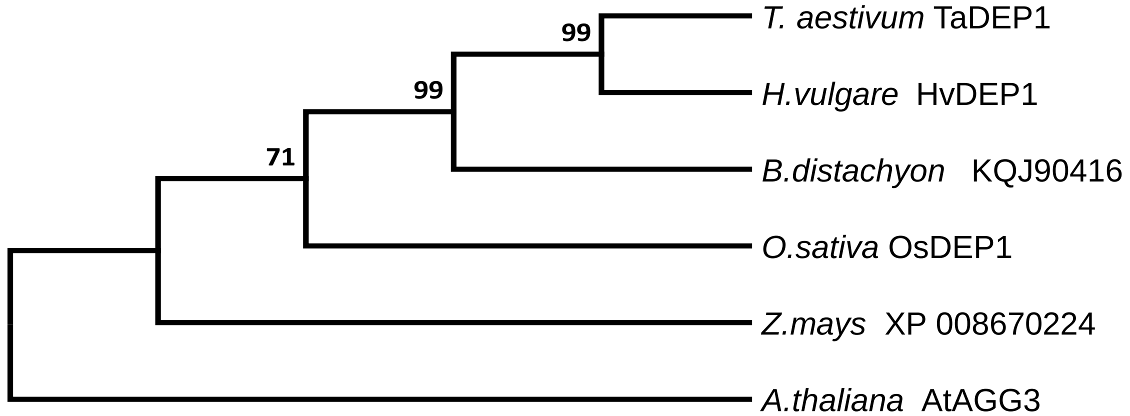

Figure S8. Phylogenetic tree of DEP1 homologs. The phylogenetic tree was constructed based on the neighbor-joining method using MEGA7 software. The evolutionary distances were computed in units of the number of amino acid substitutions per site. The GenBank accession numbers are MG758053 (TaDEP1), ACI25445 (HvDEP1), XP_015610892 (OsDEP1), NP_680175 (AtAGG3).

Figure S9

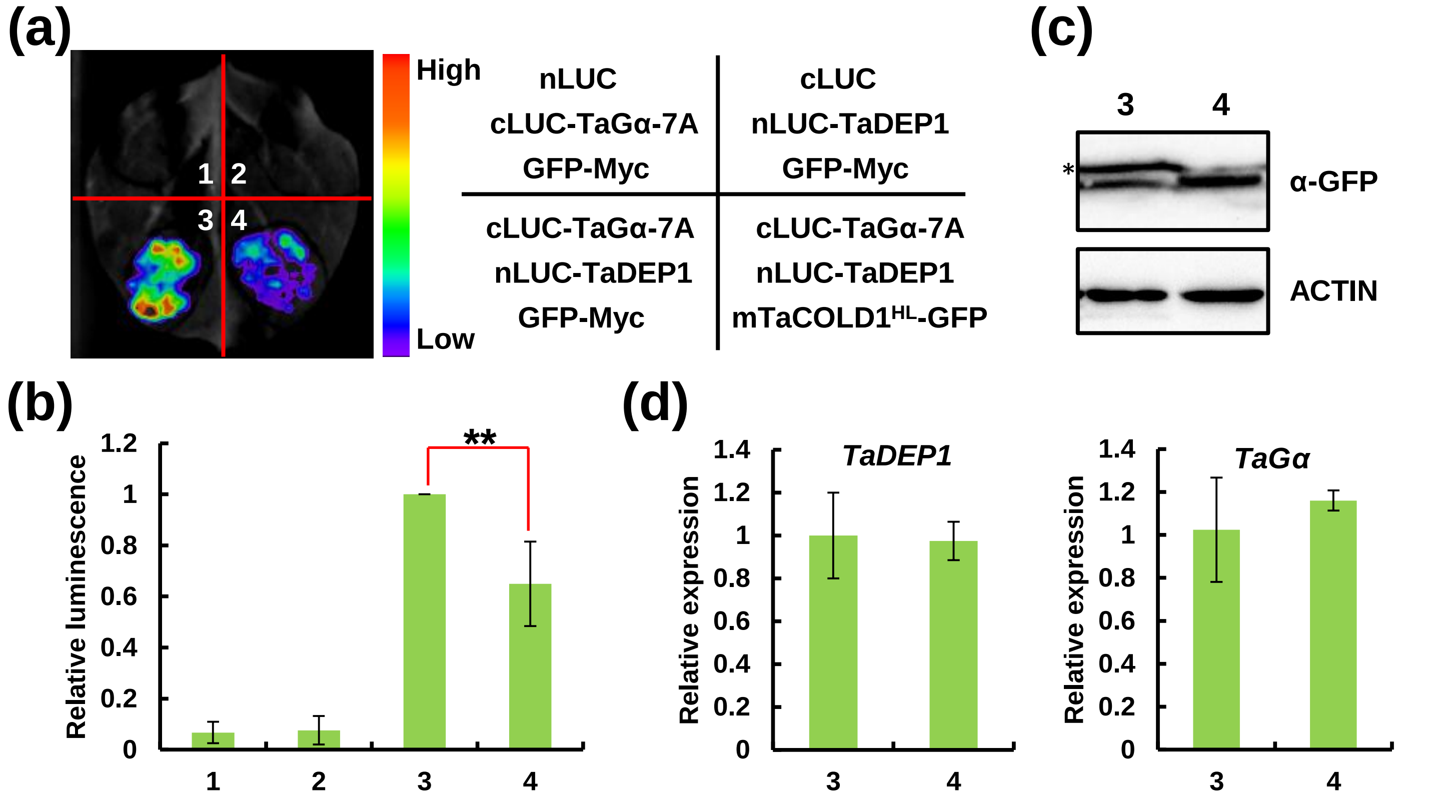

Figure S9 The mTaCOLD1 (M187K) protein interferes with the physical association between TaGα-7A and TaDEP1.

(a) LCI assay showing that the association between TaGα-7A and TaDEP1 was significantly reduced by the co-expression of mTaCOLD1. The representative image was taken at 48 hpi.

(b) Quantification of the relative luminescence intensities shown in (a), (*n*=14). The values in combination 3 were defined as "1". Error bars indicate SD among three independent replicates. **, *P*<0.01 (Student's *t* test).

(c) Immunoblot assay indicating the protein levels of mTaCOLD1-GFP and GFP-MYC in co-infiltration 3 and 4 in (a). mTaCOLD1-GFP and GFP-MYC fusion protein levels were examined with anti-GFP antibody. ACTIN was used as a loading control. Asterisk indicates the specific band.

(d) qRT-PCR assay showing the expression levels of *TaDEP1* and *TaGα* in the infiltrated *N. benthamiana* leaves shown in (a). The data were normalized to *NbACTIN1* (*NbACT1*). (Mean ± SD, n = 5).

Table S1. Conserved amino acid sequences of COLD1 homologs in rice, diploid and tetraploid wheat.

Species	Accession	Genome	Amino Acid Sequences
O. sativa COLD1*jap*			-EIDE K DIK-
O. sativa COLD1*ind*			-EIDE M DIK-
Triticum boeoticum Boiss.	BO3	A^bA^b	-EIDE M DIK-
Triticum boeoticum Boiss.	BO5	A^bA^b	-EIDE M DIK-
Triticum monococcum L.	MO5	A^mA^m	-EIDE M DIK-
Triticum urartu Thum.	UR1	A^uA^u	-EIDE M DIK-
Triticum urartu Thum.	UR205	A^uA^u	-EIDE M DIK-
Triticum urartu Thum.	UR207	A^uA^u	-EIDE M DIK-
Aegilops tauschii	Y128	DD	-EIDE M DIK-
Aegilops tauschii	Y168	DD	-EIDE M DIK-
Aegilops tauschii	Y219	DD	-EIDE M DIK-
Triticum dicoccoides Koern.	DS1	AABB	-EIDE M DIK-
Triticum dicoccoides Koern.	DS8	AABB	-EIDE M DIK-
Triticum dicoccoides Koern.	DS9	AABB	-EIDE M DIK-
Triticum dicoccoides Koern.	DS10	AABB	-EIDE M DIK-
Triticum durum Desf.	DR305	AABB	-EIDE M DIK-
Triticum durum Desf.	DR386	AABB	-EIDE M DIK-
Triticum durum Desf.	DR464	AABB	-EIDE M DIK-
Triticum durum Desf.	DR487	AABB	-EIDE M DIK-
Triticum durum Desf.	DR484	AABB	-EIDE M DIK-
Triticum turanicum Jakubz.	TR1	AABB	-EIDE M DIK-
Triticum turanicum Jakubz.	TR5	AABB	-EIDE M DIK-
Triticum carthlicum	PS6	AABB	-EIDE M DIK-
Triticum carthlicum	PS8	AABB	-EIDE M DIK-
Triticum carthlicum	PS9	AABB	-EIDE M DIK-
Tiiticum polonicum L.	PO1	AABB	-EIDE M DIK-
Triticum turgidum L.	TG2	AABB	-EIDE M DIK-
Triticum turgidum L.	TG7	AABB	-EIDE M DIK-
Triticum dicoccum L.	DM4	AABB	-EIDE M DIK-
Triticum dicoccum L.	DM18	AABB	-EIDE M DIK-
Triticum dicoccum L.	DM42	AABB	-EIDE M DIK-
Triticum dicoccum L.	DM50	AABB	-EIDE M DIK-
Triticum dicoccum L.	DM51	AABB	-EIDE M DIK-

Table S2. Primers used in this study.

Primer name	Primer sequence (5'-3')
TaCOLD1-2A-F	ATGGGGTGGGGCACGGTGGTCTAC
TaCOLD1-2A-R	ATCAATTGGATGCTTGTCTGCTT
TaCOLD1-2B-F	CACTACTCCGTCTCCGAGCCC
TaCOLD1-2B-R	GTAACCGTGTGTGGAGGTCTCCTAA
TaCOLD1-2D-F	ATGGGGTGGGGCGTGGTGGTC
TaCOLD1-2D-R	TACCACTATAATTTCCGTAACAATGT
TaCOLD1-T560A-R	GATGTCCTTTTCGTCAATTTCCCTGATAAAGAGAG
TaCOLD1-T560A-F	AAATTGACGAAAAGGACATCAAAGCTTTGGAGCG
TaCOLD1^{HL}-F	ATGAGCTACCTGTCTCTCTTTATCAGGG
TaCOLD1^{HL}-R	ATTCTGAAAATGTCCTCTCCAGGT
TaGα-7A-F	GTCATTGGCAAGCAAGGAAGC
TaGα-7A-R	ACACACAAAGTTGCGACACGAC
TaGα-1B-F	ATGTCCATGCTCGCGTGCGC
TaGα-1B-R	CAAGTTTCTGGTCCAGCGCCG
TaGα-7D-F	GTCATCTTATCTTTCAGGTCATTGG
TaGα-7D-R	TTGCCGTTTCTCTTCTCATGTG
TaGα-7AF	ATTACTTGAACCAATTCCCGATAG
TaGα-7AR	ATACTGCTTAAGAAGGAACTGCT
TaGα-1BF	CAGCTGTTGTTTGAGGACGAGAC
TaGα-1BR	TTGCTGCTCTGGAAGTAGACCTC
TaGα-7DF	TTGGACCTCTCTTCTTTAGCCTG
TaGα-7DR	AAACAGTAAAGCCCTCAAAGTGC
TaDEP1-F	ATGGGGGAGGGCGCGGTGGTGG
TaDEP1-R	TTAACACAGGCACCCGCCAGCACAG
nLUC-TaCOLD1^{HL}-F	CGGGGTACCATGAGCTACCTGTCTCTCTTTATCAGGG
nLUC-TaCOLD1^{HL}-R	ACGCGTCGACATTCTGAAAATGTCCTCTCCAGGT
cLUC-TaGα-7A-F	TACGCGTCCCGGGGCGGTACCATGTCCATGCTCGCGTGTGCGCT
cLUC-TaGα-7A-R	TCCTTGTAGTCCATTTGTTGTCACGTCCCCGTTCCTTCCCGG
cLUC-TaGα-7AΔC-R	TCCTTGTAGTCCATTTGTTGTCAGTAGATCTTGAACACCCGGTCGAC
cLUC-TaGα-1B-F	TACGCGTCCCGGGGCGGTACCATGTCCATGCTCGCGTGCGC
cLUC-TaGα-1B-R	TCCTTGTAGTCCATTTGTTGCAAGTTTCTGGTCCAGCGCCG
cLUC-TaDEP1-(NT)F	TACGCGTCCCGGGGCGGTACCATGGGGGAGGGCGCGGTG
cLUC-TaDEP1-NTR	TCCTTGTAGTCCATTTGTTGTTAGTTAATTGGTATTAGCGGGTCTT
cLUC-TaDEP1-MDF	TACGCGTCCCGGGGCGGTACCATGAAAAGGAAGCACCGGTCCTG
cLUC-TaDEP1-MDR	GTAGTCCATTTGTTGTTACGAGCCGCACTCCGGCTTG
cLUC-TaDEP1-CTF	TCCCGGGGCGGTACCATGTGTTCCGGCGGCGGCTGCT
cLUC-TaDEP1-(CT)R	TCCTTGTAGTCCATTTGTTGTTAACACAGGCACCCGCCAGCA
MBP-TaCOLD1-F	TTCAGAATTCGGATCCATGAGCTACCTGTCTCTCTTTATC
MBP-TaCOLD1-R	TTGCCTGCAGGTCGACTTAATTCTGAAAATGTCCTCTCCA
GST-TaGα-7A-F	TGGATCCCCGGAATTCATGTCCATGCTCGCGTGTGCGCT
GST-TaGα-7A-R	GGCCGCTCGAGTCGACTCACGTCCCCGTTCCTTCCCGG

Table S3. Primers used for qRT-PCR in this study.

Primer name	Primer sequence (5'-3')
TaGα-Q-F	CCCACTCCTTAACAAAGAACTCG
TaGα-Q-R	TCCAGATTCTCCATGAAGTAGTGTG
TaDEP1-Q-F	GCAGATCCTTGACCGCGAG
TaDEP1-Q-R	CACCAATAAAGACGGCAGGACC
TaCOLD1-Q-F	TGAAGCGTATAGTTGGAACAGTTGT
TaCOLD1-Q-R	CTCAAGGAATAGCTGTTTGGAAAG
TaGAPDH-Q-F	TTAGACTTGCGAAGCCAGCA
TaGAPDH-Q-R	AAATGCCCTTGAGGTTTCCC
NbACT1-Q-F	CCAAAGGCTAATCGTGAAAAG
NbACT1-Q-R	GCTGTGGTAGTGGATGAGTAAC

Table S4. Constructs used in this study.

Construct name	Vector	Description
nLUC-TaCOLD1^{HL}	p1300-35S-nLUC	LCI
nLUC-TaDEP1	p1300-35S-nLUC	LCI
nLUC-TaDEP1-NT	p1300-35S-nLUC	LCI
nLUC-TaDEP1-MD	p1300-35S-nLUC	LCI
nLUC-TaDEP1-CT	p1300-36S-nLUC	LCI
cLUC-TaGα-7A	p1300-37S-cLUC	LCI
cLUC-TaGα-1B	p1300-38S-cLUC	LCI
cLUC-TaGα-7AΔC	p1300-39S-cLUC	LCI
35S:TaCOLD1^{HL}-GFP	pGWB5	LCI
MBP-TaCOLD1^{HL}	pMAL-c2X	Pull down
GST-TaGα-7A	pGEX4T-1	Pull down
nYFP-TaCOLD1^{HL}	pEarleygate201-YN	BiFC
cYFP-TaGα-7A	pEarleygate202-YN	BiFC
nYFP-TaDEP1	pEarleygate201-YN	BiFC
pUbi:mTaCOLD1	pUbi:cas	transgenic wheat lines
